# Metabolomics and bioassay-guided characterization identifies bioactive polyphenols in *Acacia dealbata* leaf and bark extracts

**DOI:** 10.1007/s11306-026-02558-7

**Published:** 2026-11-14

**Authors:** Md Sanowar Hossain, Hemavaruni N. Fernando, Madhuri Hande, Jacqui L. Adcock, Damien L. Callahan

**Affiliations:** 1 School of Life and Environmental Sciences, Deakin University, Victoria Waurn Ponds, 3216 Australia; 2 Australian Research Council Industrial Transformation Training Centre for Facilitated Advancement of Australia’s Bioactives (FAAB), Sydney, NSW 2109 Australia; 3 Department of Pharmacy, Pabna University of Science and Technology, Pabna, 6600 Bangladesh; 4 Deakin Centre for Advanced Food Sciences, School of Exercise and Nutrition Sciences, Deakin University, Burwood, VIC 3125 Australia; 5 School of Natural Sciences, Faculty of Science and Engineering, Macquarie University, Sydney, NSW 2109 Australia; 6 School of Life and Environmental Sciences, Deakin University, 221 Burwood Highway, Burwood, Victoria 3125 Australia

**Keywords:** *Acacia dealbata*, Bioactive compounds, Untargeted metabolomics, Bioassay-guided fractionation, LC–MS/MS

## Abstract

**Introduction:**

*Acacia dealbata*, native to southeastern Australia, has documented traditional uses but remains underexplored as a source of bioactive metabolites. Comprehensive, bioassay-guided phytochemical characterization is needed to identify the bioactive compounds present in the plant.

**Objectives:**

This study aimed to identify the bioactive phytochemicals responsible for antioxidant and antimicrobial activities of *A. dealbata* leaf and bark extracts using an integrated metabolomics and natural products chemistry workflow.

**Methods:**

Leaf and bark samples were extracted using solvents of varying polarity (n-heptane, ethyl acetate, methanol, water, and water: acetonitrile: isopropanol, 2:3:3). Extracts were evaluated for antioxidant and antimicrobial activities and characterised using an LC–MS/MS-based metabolomics workflow incorporating MZmine, GNPS molecular networking, SIRIUS in silico annotation, and taxonomically informed metabolite annotation (TIMA). Bioactive extracts were fractionated by preparative HPLC, followed by bioactivity-guided assessment, quantitative LC–MS analysis using standards, and NMR spectroscopy.

**Results:**

Methanol leaf extract and ethyl acetate bark extract exhibited the strongest antioxidant and antimicrobial activities. Preparative fractionation identified flavonoids and glycosides as the dominant metabolite classes in both extracts, while terpenoids and alkaloids were also abundant in leaf fractions. Quantitative LC–MS identified rutin as the predominant compound in leaf bioactive fractions, whereas catechin, epigallocatechin, and procyanidin B2 predominated in bark fractions. NMR confirmed enrichment of flavonoid *O*-glycosides and simple phenolics in leaf fractions and flavan-3-ols and proanthocyanidins in bark fractions.

**Conclusion:**

This integrated workflow linked metabolomic profiling with bioactivity, demonstrating that *A. dealbata* leaves and bark are promising sources of bioactive polyphenols that can act against oxidative stress and infectious diseases.

**Supplementary Information:**

The online version contains supplementary material available at https://doi.org/10.1007/s11306-026-02558-7.

## Introduction

*Acacia* are widely distributed across arid, semi-arid, and temperate regions and are recognised for their ecological resilience and chemical diversity (Harris, 2004). Numerous *Acacia* species have been reported to produce a wide variety of bioactive secondary metabolites (Batiha et al., 2022). These metabolites have attracted growing interest for potential applications in food preservation, nutraceuticals, cosmetics, and pharmaceuticals (Khanam et al., 2025; Petropoulos, 2023). Within this genus, *Acacia dealbata* (Silver Wattle) is a fast-growing leguminous tree native to Southeastern Australia that has become widely naturalised in other parts of the world (Gouws & Shackleton, 2019; Hirsch et al., 2021). Despite its wide availability, *A. dealbata* remains comparatively underexplored from a comprehensive phytochemical and functional perspective.

Traditional uses in Australia by Aboriginal peoples and previous phytochemical studies on *Acacia* species have demonstrated that leaves and bark are often rich in phenolic compounds, which are closely associated with bioactivities such as antioxidant capacity and antimicrobial activity (Foreman & Walsh, 1993; House & Harwood, 1992; Uushona et al., 2021). Some studies on *A. dealbata* have indicated the presence of flavonoids, condensed tannins, and other phenolic constituents (Abilleira et al., 2021; Freire et al., 2007; Uushona et al., 2021); however, these studies have generally focused on measurement of phytoconstituents from crude extracts and only testing total phenolic contents, total flavonoid, or tannin contents (Abilleira et al., 2021; Casas et al., 2020; Lisperguer et al., 2016; López-Hortas et al., 2020; Paula et al., 2022). Therefore, a comprehensive characterization of the extracts from different tissues (specially from leaf and bark), the influence of extraction solvent polarity on metabolite recovery, identification of the chemical drivers for observed bioactivities and identification of bioactive compounds remain to be investigated.

Extraction strategy is a critical determinant of the chemical profile and therefore the bioactivity of plant extracts (Borges et al., 2020). Sequential solvent extraction using solvents of increasing polarity is a well-established approach for improving metabolite coverage and selectively enriching different classes of compounds (Palaiogiannis et al., 2023). Applying a sequential extraction strategy to the leaf and bark tissues provides an initial fractionation of the complex plant metabolome which facilitates compound identification.

Total phenolic content (TPC) and total flavonoid content (TFC) assays are commonly used as preliminary indicators of bioactive potential and are particularly useful for comparing extracts obtained using different solvents (Aryal et al., 2019; Neupane & Lamichhane, 2020). However, these assays provide only aggregate measurements and do not identify the individual compounds responsible for biological activity (Abilleira et al., 2021; Casas et al., 2020; Lisperguer et al., 2016; Paula et al., 2022). Comprehensive chemical characterization is therefore essential to move beyond bulk measurements to better understand metabolite–bioactivity relationships.

Liquid chromatography–tandem mass spectrometry (LC–MS/MS) based untargeted metabolomics has become an indispensable tool for the comprehensive profiling of complex plant extracts (Dadwal et al., 2026; Kilinc et al., 2023). Recent advances in computational metabolomics have substantially improved the interpretability of LC–MS/MS data by enabling more robust feature detection, spectral alignment, and metabolite annotation across complex biological samples (Cai et al., 2023). Modern data-processing workflows allow thousands of molecular features to be deconvoluted from raw LC–MS data, aligned across samples to compare, and provides a comprehensive overview of chemical diversity (Cai et al., 2023; Yao et al., 2019). Feature-based molecular networking (FBMN) further enhances this process by organising MS/MS spectra into molecular networks based on MS/MS spectral similarity, thereby grouping structurally related metabolites and facilitating the visual exploration of chemical families, isomers, and fragmentation patterns. This network-based representation is particularly valuable for plant metabolomics, where large numbers of structurally related secondary metabolites are often present (Nothias et al., 2020; Yao et al., 2019).

In silico annotation tools such as SIRIUS have further advanced metabolite identification by enabling the prediction of molecular formulas and candidate structures directly from high-resolution MS/MS spectra (Dührkop et al., 2019, 2021). Such capabilities are especially important for natural product research, where many metabolites are novel or poorly represented in public spectral libraries (Dührkop et al., 2019, 2021).

Taxonomically informed metabolite annotation (TIMA) represents an additional layer of improvement in annotation by incorporating biological and taxonomic knowledge into the process (Rutz et al., 2019). TIMA leverages known metabolite distributions within taxonomic lineages to re-rank chemically plausible annotations, thereby reducing false positives and improving biological relevance. By constraining candidate annotations to metabolites previously reported within related taxa, TIMA enhances annotation confidence while simultaneously highlighting potentially novel or lineage-specific compounds (Allard et al., 2016; Rutz et al., 2019).

Collectively, the integration of FBMN, SIRIUS, and TIMA enables a multi-layered interpretation of untargeted LC–MS/MS data (Rutz et al., 2019). This combined strategy allows not only the annotation of individual metabolites but also the characterization of broader chemical families (Rutz et al., 2019). For complex and insufficiently characterised species like *A. dealbata*, such integrative computational workflows are essential for effectively linking metabolite profiles with biological activity.

Bioassay-guided fractionation remains a fundamental tool in natural product research for identifying biologically active constituents (Das & Shafi, 2023). The integration of untargeted metabolomics with bioassay-guided fractionation offers a more efficient and informative approach, enabling the prioritisation of extracts and fractions based on both chemical features and biological activity (Mani et al., 2024). By iteratively linking metabolomic profiles with bioassay outcomes, this strategy facilitates the selection of bioactive extracts or fractions, then identification of compounds or compound classes that contribute to observed activities (Mani et al., 2022, 2024).

Antioxidant and antimicrobial activities are particularly relevant for evaluating the functional potential of *A. dealbata* extracts, especially considering their documented traditional uses (Foreman & Walsh, 1993; House & Harwood, 1992; Williams & Sides, 2008; Zola & Gott, 1992). Given the increasing demand for natural antioxidants and alternative antimicrobial agents, systematic evaluation of these activities in *A. dealbata* extracts highlights their potential for application in food, nutraceutical, and pharmaceutical contexts from a fast growing and widely distributed species world-wide.

Overall, this work provides a systematic and in-depth assessment of *A. dealbata* leaf and bark as a source of bioactive phytochemicals and demonstrates the value of combining untargeted metabolomics and bioassay-guided fractionation for natural product research. The findings contribute new insights into tissue-specific chemistry of *A. dealbata*, their detailed chemical characterization, and metabolite–bioactivity relationships, while also highlighting the potential of *A. dealbata* leaf and bark as underutilised natural resources.

## Methodology

### Extraction from *A. dealbata* leaf and bark

#### Sample collection and preparation

*A. dealbata* leaf and bark samples were collected from trees in Geelong, Victoria. Samples were collected fresh, in the morning, sorted and stored immediately at -20 °C before freeze drying. Bark samples were cut into small pieces for effective drying and grinding. Approximately 800 g of each sample were dried in a laboratory scale freeze dryer (Christ, Germany), then ground using a coffee grinder with liquid nitrogen. Finally, powdered samples were stored in -20 °C freezer until used for extraction.

#### Sample extraction

Powdered leaf and bark samples (100 g each) were subjected to two extraction methods: sequential and single solvent extraction. For sequential extraction, samples were extracted successively with *n*-heptane, ethyl acetate, methanol, and deionized water (1:5, w/v). For each solvent, 500 mL was added to the plant material in conical flasks, ultrasonicated for 30 min, and stirred for an additional 30 min at room temperature. The mixture was centrifuged at 4000 rpm for 10 min, and supernatants were collected. This process was repeated twice, and the pooled extracts were concentrated using a rotary evaporator then taken to dryness under nitrogen gas, while aqueous extracts were freeze-dried. Between solvent steps, the residual biomass was dried overnight at room temperature.

For single solvent extraction, 100 g of the same plant material was extracted with a mixture of water: acetonitrile: isopropanol (2:3:3, v/v/v) using the same sonication, stirring, and centrifugation steps. Three successive extractions were performed, and the combined supernatants were concentrated using a rotary evaporator, then freeze-dried to obtain the final extract. All dried extracts were weighed, and extraction yields were calculated.

### Measurement of phytoconstituents

#### Determination of total phenolic content (TPC)

The total phenolic content (TPC) of the extracts was determined using the Folin–Ciocalteu colorimetric method, as described by Singleton and Rossi (Singleton & Rossi, 1965), with slight modifications. Gallic acid was used as the standard. Sample solutions were prepared at 250 µg/mL concentrations, while gallic acid standards were prepared at 0 to 100 µg/mL. To each test tube, 0.5 mL of the sample or standard solution was added, followed by 2.5 mL of 2% (v/v) Folin–Ciocalteu reagent. The mixture was left to react at room temperature for 5 min. Then, 2 mL of 7.5% (w/v) sodium nitrite (NaNO₂) solution was added, and the reaction mixtures were incubated in dark for 90 min. Blanks were prepared by replacing the sample or standard solution with deionised water (18.2 MΩ, Milli-Q) water while maintaining the same reagent volumes and conditions. Absorbance was measured at 760 nm using a UV–Vis spectrophotometer (Cary 300 UV-Vis, Agilent Technologies, Australia). All measurements were conducted with three technical replicates from a pooled biological sample, prepared and analysed separately. The TPC was calculated from the gallic acid standard curve (Supplementary Fig. 1) and expressed as milligrams of gallic acid equivalents per gram of extract.

#### Determination of total flavonoid content (TFC)

The total flavonoid content (TFC) of the extracts was determined using the aluminium chloride colorimetric method, as described by Chang et al. (Chang et al., 2002), with slight modifications. Quercetin was used as the standard. Sample solutions (250 µg/mL) and quercetin standards (20 to 200 µg/mL) were prepared, and 0.5 mL of each was transferred into separate test tubes. Then, 0.3 mL of 5% (w/v) sodium nitrite (NaNO₂) solution was added to each tube, and the mixture was incubated at RT for 5 min. Subsequently, 0.3 mL of 2% (w/v) aluminium chloride (AlCl_3_) solution was added and allowed to react for 6 min. Then, 2 mL of 1 M sodium hydroxide (NaOH) was added, followed by 1.9 mL of deionized water to make the final volume to 5 mL and were mixed thoroughly. A blank was prepared by replacing the sample or standard with deionized water while adding all other reagents in the same sequence and volume. Absorbance was measured at 506 nm using a UV–Vis spectrophotometer. All measurements were conducted with three technical replicates and the TFC was determined from the quercetin standard calibration curve (Supplementary Fig. 1) and expressed as milligrams of quercetin equivalents (mg QE) per gram of extract.

### Assay of bioactivities

#### Antioxidant activity assay

##### DPPH radical scavenging assay

The free radical scavenging activity of the extracts and fractions was evaluated by DPPH (2,2-diphenyl-1-picrylhydrazyl) assay using the method described by Brand-Williams et al. (Brand-Williams et al., 1995). For the assay, samples and standard L-ascorbic acid were taken in different concentrations and diluted to 2.5 mL with methanol to make 10–160 µg/mL final concentration. Then 2.5 mL of the 0.01mM DPPH solution in methanol was added to each tube and incubated in the dark for 30 min. A control was prepared by adding methanol and DPPH solution in 1:1 ratio and incubated under the same conditions. Finally, absorbance of control, standard and samples were measured at 517 nm in a UV–Vis spectrophotometer using methanol as a blank.

For fraction analysis, the same method was applied using a microplate reader. Samples and standard L-ascorbic were initially prepared at a stock concentration of 100 µg/mL and then diluted to final concentrations of 5, 10, 20, 40, and 60 µg/mL in a total volume of 100 µL per well. Subsequently, 100 µL of 0.1 mM DPPH solution was added to each well. All samples were analysed with three technical replicates. The plates were incubated in the dark for 30 min, and absorbance was measured at 517 nm against a blank using a microplate reader (Thermo Fisher Scientific, Australia).

DPPH radical scavenging activity was calculated by using the following equation:

$$\% \text{DPPH scavenging} = \left( \frac{\text{Absorbance of control} - \text{Absorbance of sample}}{\text{Absorbance of control}} \right) \times 100$$

##### Ferric reducing antioxidant power (FRAP) assay

The FRAP assays of the samples and fractions followed the method of Oyaizu (Oyaizu, 1986). For the assay, 1 mL of the samples and standard L-ascorbic acid was prepared at different concentrations (12.5–200 µg/mL) and transferred to test tubes, followed by the addition of 1 mL phosphate buffer (pH = 6.6). Then 1 mL of 1% (w/v) potassium ferricyanide [K₃Fe(CN)₆] was added to the tubes and incubated for 20 min at 50 °C in a convection oven. After incubation, 1 mL of 10% (w/v) trichloroacetic acid (TCA) was added to stop the reaction. Tubes were centrifuged at 2400 rpm for 10 min, and 2 mL of the supernatants were transferred into new tubes and diluted with 2 mL deionized water. Finally, 1 mL of the 0.1% (w/v) ferric chloride (FeCl₃) solution was added to each tube, mixed, and absorbance was measured using a UV–Vis spectrophotometer at 700 nm against the blank. Blank was prepared by following the same method replacing the sample with deionized water.

For fraction analysis, the same method was used in a microplate scale. Samples and standard L-ascorbic were prepared at a stock concentration of 200 µg/mL and diluted to final concentrations of 12.5, 25, 50, 100, and 200 µg/mL in Eppendorf tubes, with the total volume adjusted to 100 µL. To each tube, 100 µL of phosphate buffer (pH 6.6) and 100 µL of 1% (w/v) potassium ferricyanide solution were added, and the mixtures were incubated at 50 °C for 20 min in an oven. The reaction was stopped by adding 100 µL of 10% (w/v) TCA, followed by centrifugation at 2400 rpm for 10 min. Subsequently, 80 µL of the supernatant was transferred to a 96-well microplate and diluted with 80 µL of deionized water, after which 40 µL of 0.1% (w/v) ferric chloride solution was added to each well. Absorbance was measured at 700 nm using a microplate reader.

All samples were analysed with three technical replicates, and the reducing power of the samples was calculated based on the standard curve constructed from L-ascorbic acid (Supplementary Fig. 3 and Supplementary Fig. 4) and expressed as ascorbic acid equivalents (µg/mL of extract).

##### Total antioxidant capacity assay

The total antioxidant activity of the extracts and fractions was determined using the phosphomolybdenum assay as described by Prieto et al. (Prieto et al., 1999). Briefly, 1 mL of each sample or L-ascorbic acid standard solution (5 to 60 µg/mL) was mixed with 1.5 mL of reagent solution containing sulfuric acid, sodium phosphate monobasic, and ammonium molybdate. The mixtures were incubated at 95 °C for 90 min, then cooled to room temperature. The absorbance was measured at 695 nm against a blank using a UV–Vis spectrophotometer. The blank was prepared by replacing the sample or standard with methanol and treated identically.

Similar to the other assays, fractions and the standard L-ascorbic acid were analysed at the microplate scale. Samples were prepared at a stock concentration of 100 µg/mL and diluted in the microplate wells to final concentrations of 5, 10, 20, 40, and 60 µg/mL, with the total volume adjusted to 100 µL. Subsequently, 100 µL of the reagent solution was added to each well. The reaction mixtures were incubated at 95 °C for 90 min in an oven, cooled to room temperature, and the absorbance was measured at 695 nm against a blank containing methanol and reagent only.

A standard calibration curve was constructed using L-ascorbic acid, and the total antioxidant capacity of the samples was expressed as L-ascorbic acid equivalents, calculated from the slope of the standard curve (Supplementary Fig. 5 and Supplementary Fig. 6). All samples were analysed with three technical replicates.

#### Antimicrobial activity assay

The antimicrobial activity of *A. dealbata* extracts and fractions was evaluated using the agar disc diffusion method, following the principles of the Kirby–Bauer assay (Bauer et al., 1966) with minor modifications. Test microorganisms included two Gram-positive bacteria (*Geobacillus* sp. and *Staphylococcus aureus*), two Gram-negative bacteria (*Escherichia coli* and *Pseudomonas aeruginosa*), and one yeast (*Saccharomyces cerevisiae*). Fresh microbial cultures were prepared prior to each assay and adjusted to a turbidity equivalent to 0.5 McFarland standard (approximately 1.5 × 10⁸ CFU/mL).

Standardised bacterial suspensions were uniformly spread onto Mueller–Hinton agar (MHA) plates, while yeast suspensions were spread onto potato dextrose agar (PDA) plates using sterile cotton swabs. Sterile paper discs were impregnated with 100 µg of extract per disc (10 µL from a 10 mg/mL stock solution), allowed to dry under aseptic conditions, and placed onto the inoculated agar surfaces. All assays were performed with three technical replicates. Chloramphenicol (30 µg/disc) was used as the positive control for bacterial strains, and fluconazole (20 µg/disc) was used as the positive control for yeast. Plates were incubated under strain-specific conditions: *Geobacillus* sp. at 55 °C, other bacterial strains at 37 °C for 24 h, and *S. cerevisiae* at 25 °C for 48 h. Following incubation, antimicrobial activity was assessed by measuring the diameter of the zone of inhibition (mm) surrounding each disc. Mean inhibition zones and standard deviations were calculated from triplicate measurements. Based on the initial screening results of crude extracts, *E. coli* was excluded from subsequent assays of leaf and bark fractions, which were evaluated using the same experimental protocol.

### Fractionation of the extracts

The selected extracts were fractionated using a Thermo Fisher UltiMate 3000 series HPLC system (Thermo Fisher Scientific, Australia) with diode array UV-Vis detector and equipped with a GRACE Vision C18 Classic column (5 μm, 22 × 250 mm) and an automated fraction collector. The mobile phase consisted of solvent A deionised water (18.2​ MΩ, Milli-Q) with 0.1% formic acid and solvent B acetonitrile (LC-MS grade; Thermo Fisher Scientific, Australia) with 0.1% formic acid. Gradient elution was performed using an optimized mobile phase composition and flow rate. The flow rate was set at 8.0 mL/min, starting with 10% B, gradually increasing to 100% B over 40 min, holding at 100% B until 47 min, then decreasing sharply to 5% B and continuing until the run ended at 55 min.

The leaf methanol extract (L-MET) was dissolved in methanol, and the bark ethyl acetate extract (B-ETA) was dissolved in methanol: isopropanol (1:1) to prepare 20 mg/mL stock solutions. The injection volume for each run was 500 µL. UV–visible detection was performed at four wavelengths (215, 240, 280, and 380 nm) and the full spectrum from 200 to 500 nm to monitor eluting peaks. For the L-MET sample, fractions were collected based on the peaks detected at 380 nm. The automated fraction collector dispensed individual peaks into separate tubes, and equivalent fractions from replicated injections were pooled, resulting in a total of 17 fractions (Supplementary Table 2). For the B-ETA sample, time-based fractionation was applied, with fractions collected every 5 min from 5 to 55 min, yielding a total of 10 fractions (Supplementary Table 2). Fractions collected at the same time intervals from subsequent runs were pooled together. All pooled fractions were concentrated using rotary evaporation at 30 °C to remove acetonitrile and reduce volume, then frozen at − 80 °C and lyophilized. The dried fractions were weighed in pre-weighed tubes and stored at -80 °C for further bioassay and chemical characterization.

### Phytochemical characterization

#### LC–MS analysis of the sample

Extracts and fractions were analysed using a Vanquish UHPLC system coupled to a high-resolution Orbitrap Exploris 240 mass spectrometer (Thermo Fisher Scientific, Australia) equipped with a Waters Acquity UPLC C18 column (1.7 μm, 2.1 × 150 mm). Dried extracts were dissolved in methanol, except for aqueous extracts, which were dissolved in deionized water, to a concentration of 3 mg/mL. Solutions were filtered through 0.45 μm syringe filters and transferred into 2 mL HPLC vials. Fraction samples were prepared at 1 mg/mL.

For each run, 1 µL of sample was injected for a 30-minute analysis using a binary gradient at a flow rate of 0.4 mL/minute. Mobile phase A consisted of 0.1% (v/v) formic acid in water (LC–MS grade, Fisher Scientific, Australia), and mobile phase B was 0.1% (v/v) formic acid in LC–MS grade acetonitrile (Fisher Scientific, Australia). The gradient began at 5% B, increased linearly to 100% B over 20 min, held for 6 min, then returned to 5% B to re-equilibrate the column. The column temperature was maintained at 30 °C.

Mass spectrometric analysis was performed in both positive and negative electrospray ionization (ESI) modes, acquiring both MS¹ and MS² spectra. Data-dependent acquisition (DDA) was used with a mass range of *m/z* 80–1200, a resolution of 120,000, and RF lens setting of 70%. Higher-energy collisional dissociation (HCD) was applied with normalized collision energies of 30, 60, and 80%. Easy-IC™ mass calibration was used to keep mass error below 2 ppm. Data were recorded in centroid mode and subsequently processed using metabolomics software tools.

For quantitative LC–MS, a mixture of 11 standards comprising the polyphenols catechin, epigallocatechin, quercetin, myricetin, luteolin, naringenin, apigenin, genistein, rutin, isorhamnetin, and procyanidin B2 was used. These compounds were quantified in L-MET, B-ETA, and in the fractions using negative ionization mode in LC–MS. The standard mix was prepared at eight different concentrations (from 0.05 to 20 µM) by dissolving the required amounts in methanol, while the fractions were prepared in 0.5 mg/mL, and extracts (L-MET and B-ETA) were prepared in 1.0 mg/mL concentrations. Injection volume was 1 µL for all samples and standards.

#### NMR-based analysis of the bioactive fractions

Based on the bioassays and quantities obtained after fractionation, 6 fractions were selected for NMR-based characterization. Samples were analysed using solution-state nuclear magnetic resonance (NMR) spectroscopy for structural characterization of the major metabolite classes present in the extracts. Bark fractions were dissolved in CD_3_OD, whereas the leaf fraction was dissolved in DMSO-d_6_ selected according to solubility characteristics to ensure complete sample dissolution prior to NMR analysis.

All NMR experiments were acquired on a Bruker Avance III HD 500 MHz spectrometer equipped with a room-temperature 5 mm BBFO probe at 298 K at the Macquarie University Centre for Analytical Biotechnology and NMR (MUCAB/NMR), Macquarie University. One-dimensional ^1^H, and ^13^C NMR spectra were recorded for initial metabolite profiling. Two-dimensional NMR experiments including COSY, multiplicity-edited HSQC and HMBC were subsequently acquired to investigate proton-proton and proton-carbon correlations within the complex mixtures (Supplementary Fig. 10). Representative acquisition parameters and additional experimental details are provided in the Supplementary Table 7.

Multiplicity-edited HSQC spectra were acquired using the Bruker pulse sequence *hsqcedetgpsisp2.3* to distinguish CH/CH_3_ and CH_2_ resonances based on signal phase. HMBC spectra were acquired using a long-range ^n^$\:{J}_{CH}$coupling constant of 10 Hz to detect two- and three-bond heteronuclear correlations.

### Data analysis

#### LC-MS data analysis

LC–MS/MS data were analysed using a comprehensive untargeted metabolomics workflow integrating advanced data processing tools including MZmine (v4.8.0), GNPS2, SIRIUS (v6.3.0), and the taxonomically informed metabolite annotation (TIMA) approach in R (Rutz et al., 2019).

Centroid LC–MS data (.raw) were first converted to indexed mzML files using ThermoRawFileParser (v1.4.5). The mzML files were then processed in MZmine for feature detection, alignment, and quantification. For each sample, triplicate runs were aligned, and background noise was subtracted using respective blanks. Peak detection and alignment parameters included smoothing, stable ionization across samples, minimum MS¹ peak intensity of 5 × 10⁶, MS² intensity threshold of 2.5 × 10⁰, noise level of 5 × 10⁵, peak duration range of 0.05–0.5 min, and a minimum of one aligned feature per sample. Considered adducts in positive mode included [M + H]⁺, [M + 2 H]²⁺, [M + K]⁺, [M + Na]⁺, [M + NH₄]⁺, and [M + ACN]⁺ with corresponding negative adducts in negative ionization mode. MZmine outputs were then used for downstream analyses in SIRIUS, GNPS2, and TIMA.

For feature-based molecular networking (FBMN) in GNPS2, the preset FBMN workflow was applied using the MZmine-generated feature list, spectra file, and supplementary edge information. Compound annotation employed the GNPS spectral library with a minimum cosine score of 0.7 and at least six matched peaks. The resulting molecular networks were visualized in Cytoscape (v3.10.3) to identify chemical clusters, compare samples, and highlight potential bioactive compounds. Additional outputs from MZmine were imported into SIRIUS (v 6.1.1) for in silico molecular formula determination, structure elucidation, and compound class prediction. SIRIUS also utilized its integrated databases to enhance annotation confidence (Dührkop et al., 2019).

Finally, TIMA in R was applied to integrate MS data, re-rank SIRIUS predictions, and refine metabolite annotations using taxonomic and GNPS metadata. TIMA uses natural product libraries such as COCONUT and LOTUS to associate chemical features with known taxa (family, genus, or species), improving annotation accuracy across the dataset (Rutz et al., 2019). Positive and negative ionization data from LC–MS were processed separately, and final TIMA outputs were merged using a Python script based on the *m/z* values of the features. The combined dataset was further analysed in Python to generate sunburst plots and bar graphs comparing compound superclasses, classes, and subclasses.

For targeted LC–MS analysis of standard polyphenols in crude extracts and fractions, data processing, compound identification, and quantification were performed using Thermo TraceFinder 5.1 General LC software. Compounds were identified based on their MS1 chromatogram and retention times by comparing with the standards. For quantification, a standard curve was constructed for each compound (with a minimum R^2^ value of 0.998), and concentrations of the compounds in extracts and fractions were determined.

#### NMR data analysis

All spectra were processed and analysed using Bruker TopSpin 4.5.0 software. Peak picking for the ^1^H and ^13^C spectra was performed automatically and subsequently verified by manual inspection, while COSY, HSQC and HMBC peak lists were generated manually to minimise artefacts arising from spectral overlap and low-intensity noise signals in the complex crude extracts. Diagonal peaks were excluded from the final COSY peak lists used for spin-system extraction. Low-intensity and overlapping resonances were interpreted conservatively, and only reproducible correlations were used for class-level structural annotation.

Tentative spin systems were extracted to assist structural interpretation of the fractions using the NMRMine Python workflow, which integrates manually curated COSY and HSQC peak lists to construct proton connectivity networks and assign associated ^13^C chemical shifts. The generated spin systems were subsequently evaluated manually, as some automatically connected networks arose from spectral overlap, noise correlations, and merged resonances commonly encountered in complex natural product mixtures. The final NMRMine-derived spin systems are provided in the Supplementary Table 8. The NMRMine workflow is described with the supplementary table and was used solely as an organisational tool for class-level interpretation; it does not provide automated compound identification or definitive structural assignments. Selected spectra were processed and visualized in MestReNova for figure preparation and presentation.

Structural interpretation focused on identifying characteristic chemical shift patterns and connectivity motifs associated with polyphenolic natural products, including flavan-3-ols, proanthocyanidins, flavonoids, flavonoid glycosides and phenolic acids. The NMR observations were subsequently compared with tentative metabolite annotations obtained from GNPS-based LC–MS/MS analysis to support class-level consistency between the two analytical approaches.

## Results and discussion

### Extraction from leaf and bark

Sequential and single-solvent extraction of leaf tissues of *A. dealbata* yielded 1.72–14.23% (w/w) of dry plant material. Among the solvents tested, n-heptane produced the lowest extraction yield, whereas methanol afforded the highest. Extraction using the mixed solvent system [water: acetonitrile: isopropanol, 2:3:3 (WAI)] generated a yield comparable to that obtained with methanol. Similarly, extraction of bark tissues produced 0.61–20.85% (w/w) yields, with n-heptane again giving the lowest extract mass and the mixed solvent system providing the highest yield (Table 1).

**Table 1:** Percentage yield of the extracts using different solvents from leaf and bark

Sample name	% Yield	Sample name	% Yield
*A. dealbata* leaf samples		*A. dealbata* bark samples	
L-NHP	1.72	B-NHP	0.61
L-ETA	3.28	B-ETA	4.09
L-MET	14.2	B-MET	15.7
L-AQU	5.28	B-AQU	6.37
L-WAI	13.7	B-WAI	20.9

Here, *L*-, and *B*- represent leaf and bark extracts respectively, *NHP* n-heptane extract, *ETA* ethyl acetate extract, *MET* methanolic extract, *AQU *aqueous extract, and WAI  extract using water: acetonitrile: isopropanol (2:3:3), values are calculated on a dry weight basis of the sample

The low yield observed with n-heptane, a highly non-polar solvent, is consistent with its preferential extraction of lipophilic constituents such as lipids, fatty acids, waxes, and other non-polar components from plant tissues (Saini et al., 2021). The use of non-polar solvents to remove such lipidic materials is a common defatting step in phytochemical extraction, which helps reduce extract complexity and facilitates subsequent isolation or analysis of secondary metabolites and reduces the need for strong solvents when using reversed phase chromatography (Hossain et al., 2016; Starmans & Nijhuis, 1996). Consequently, the n-heptane extracts were excluded from further analysis, and the remaining four extracts from both leaf and bark tissues were selected for subsequent biological activity evaluation and chemical characterization.

### Total phenolic and flavonoid content

The total phenolic content (TPC) and total flavonoid content (TFC) of leaf and bark extracts of *A. dealbata* were quantified using colorimetric assays expressed as gallic acid (GA) and quercetin (QCT) equivalents (Table 2 and Supplementary Table 1). Considerable variation in both TPC and TFC was observed among extracts depending on solvent type and plant tissue.

**Table 2:** Quantitative measurement of phenolic and flavonoid content in *A. dealbata* tissues using colorimetric methods

Sample	Total phenolic content [GA equivalent concentration (mg/g)]	Total flavonoid content [QCT equivalent concentration (mg/g)]
L-ETA	229 ± 3^b^	129 ± 9^b^
L-MET	391 ± 5^a^	211 ± 14^a^
L-AQU	167 ± 3^b^	47 ± 12^b^
L-WAI	426 ± 2^a^	213 ± 24^a^
B-ETA	567 ± 3^c^	434 ± 9^d^
B-MET	473 ± 8^c^	311 ± 29^c^
B-AQU	328 ± 2^d^	188 ± 3^d^
B-WAI	492 ± 4^c^	327 ± 13^c^

Here, values are mg/g of extract. *GA* gallic acid, *QCT* quercetin, values are mean ± standard deviation, *n* = 3. For leaf extracts, different superscript letters (a, b) indicate significant differences from L-WAI based on pairwise two-tailed Welch’s t-tests with Bonferroni correction (adjusted α = 0.0167). For bark extracts, different superscript letters (c, d) indicate significant differences from B-WAI using the same statistical criteria. Samples sharing a letter are not significantly different

Among the leaf extracts, the mixed solvent extract (L-WAI) showed the highest TPC, followed by the methanol extract (L-MET), and these two extracts were not significantly different from each other (*p* < 0.01). Both extracts contained significantly higher phenolic levels than the ethyl acetate (L-ETA) and aqueous (L-AQU) extracts. A similar trend was observed for flavonoid content, where L-WAI and L-MET exhibited substantially higher TFC than L-ETA and L-AQU. These results indicate that polar organic solvents or mixed solvent systems are more effective for extracting phenolic and flavonoid compounds from leaf tissues. Although the mixed solvent extract and the methanolic extract showed similar TPC and TFC, a direct comparison between them is not appropriate due to differences in the extraction workflows. The mixed solvent extract was obtained through a single-step extraction process, whereas the methanolic extract was derived from samples that underwent sequential solvent treatment, including pre-extraction with n-heptane and ethyl acetate prior to methanol extraction. Therefore, with comparable TPC and TFC values, the methanolic extract demonstrated superior overall performance relative to the mixed solvent extract.

For bark extracts, both TPC and TFC were generally higher than those observed in leaves. The highest phenolic content was detected in B-ETA, followed by B-WAI and B-MET, while B-AQU showed significantly lower values. Similarly, B-ETA exhibited the highest flavonoid content, followed by B-WAI and B-MET, whereas B-AQU contained the lowest amount.

The markedly higher phenolic and flavonoid contents in bark extracts suggest that bark tissues of *A. dealbata* are a richer source of polyphenolic secondary metabolites than leaves. Bark from different *Acacia* species has also been reported as a rich source of polyphenols, particularly flavonoids and condensed tannins (Elshamy et al., 2024). Bark serves as the permanent outer protective layer of plants, enduring prolonged exposure to environmental stressors and defending against wood-boring insects, fungal pathogens, and large herbivores (Häsler Gunnarsdottir et al., 2023). In this context, phenolic compounds and flavonoids, especially condensed tannins function as chemical deterrents and antimicrobial agents that contribute to plant defence and survival. Consistent with these functions, the present findings shows that total phenolic content (TPC) and total flavonoid content (TFC) in *A. dealbata* is higher than some other *Acacia* species such as *A. seyal*, *A. mearnsii*, and *A. nilotica* (TPC 192–452 mg GAE/g, TFC 59–200 mg CE/g) (Elmi et al., 2020; Foyzun et al., 2022). Overall, these results highlight the potential bioactivity of *A. dealbata* tissues, particularly bark extracts, as phenolics and flavonoids are key secondary metabolites widely associated with diverse biological activities (Dai & Mumper, 2010; Panche et al., 2016).

### Assay of the in vitro antioxidant activity

#### DPPH radical scavenging assay

**Fig. 1:**
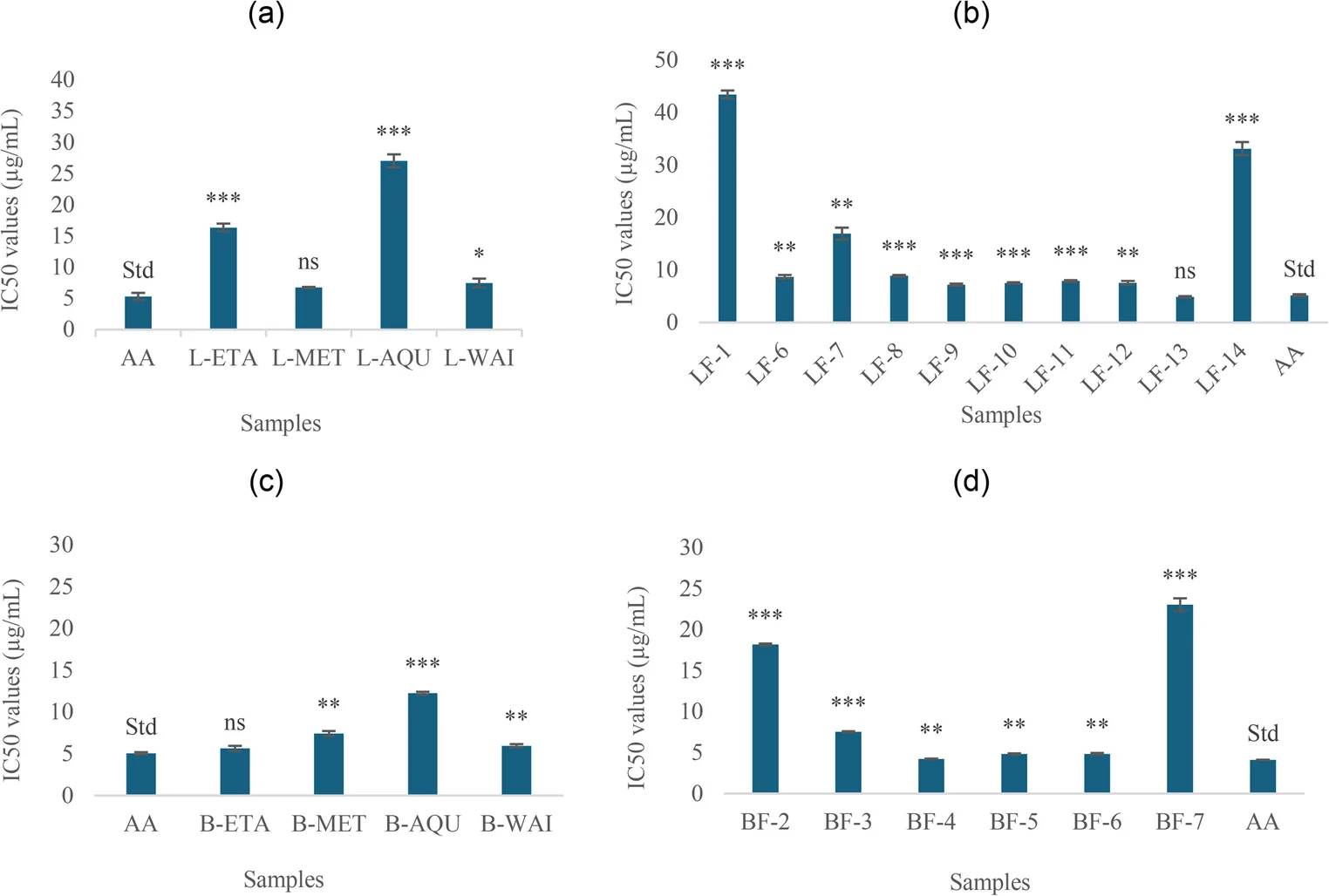
Inhibitory concentration 50 (IC_50_) values of the extracts and fractions against 0.1 mM DPPH solution. Figure (**a**) represents values for leaf extracts, (**b**) represents leaf active fractions, (**c**) represents bark extracts, and (**d**) represents bark active fractions. Other fractions not shown in the graph (LF-2, LF-3, LF-4, LF-5, LF-15, LF-16, LF-17, BF-1, BF-8, BF-9, BF-10) showed very low antioxidant activity with IC_50_ value > 60. AA = standard L-ascorbic acid. LF = leaf fractions from methanolic extract (L-MET), BF = bark fractions from ethyl acetate extract (B-ETA). Statistical analysis was performed using an unpaired two-tailed Welch’s t-test to compare samples with AA. Data are expressed as mean ± SD (*n* = 3). Differences were considered significant at *p* < 0.05. *p < 0.05 **p < 0.01, ***p < 0.001, ns = difference is not significant (*p* ≥ 0.05)

The antioxidant potential of the extracts from *A. dealbata* tissues was evaluated using the DPPH radical scavenging assay. The results revealed notable variations in radical scavenging activity among the different solvent extracts, indicating that both plant tissue type and extraction solvent influenced the antioxidant capacity (Fig. 1 and Supplementary Fig. 2). Among the leaf extracts, L-MET and L-WAI showed a significantly potent antioxidant activity, especially L-MET showed no significant difference (*p* ≥ 0.05) with the standard L-ascorbic acid (AA) (Fig. 1a). The IC_50_ value for both extracts was significantly lower than the ethyl acetate and aqueous extracts. For the bark extracts, the ethyl acetate extract (B-ETA) showed similar potent antioxidant activity to the standard L-ascorbic acid, followed by a similar activity by the mixed solvent extract (B-WAI) (Fig. 1c).

Based on the initial bioassays, L-MET and B-ETA were selected for further fractionation using preparative HPLC and the subsequent fractions were tested for free radical scavenging activity using similar methods. Among the seventeen leaf fractions, seven fractions (LF-6, LF- 8, LF- 9, LF- 10, LF- 11, LF- 12, LF- 13) showed potent DPPH radical scavenging activity (with IC_50_ values < 9 µg/mL), where LF-13 was most active with no significant difference with the standard L-ascorbic acid (Fig. 1b). Similar to leaf fractions, bark ethyl acetate extract (B-ETA) was fractioned into ten different fractions and tested in the radical scavenging assay where BF-4, BF-5, and BF-6 showed the most potent radical scavenging activity when compared to L-ascorbic acid (*p* < 0.01) (Fig. 1d). The potent DPPH radical scavenging activity of these fractions indicated their potential bioactivity and were chosen for further investigation.

#### Ferric reducing antioxidant power (FRAP) assay

**Fig. 2:**
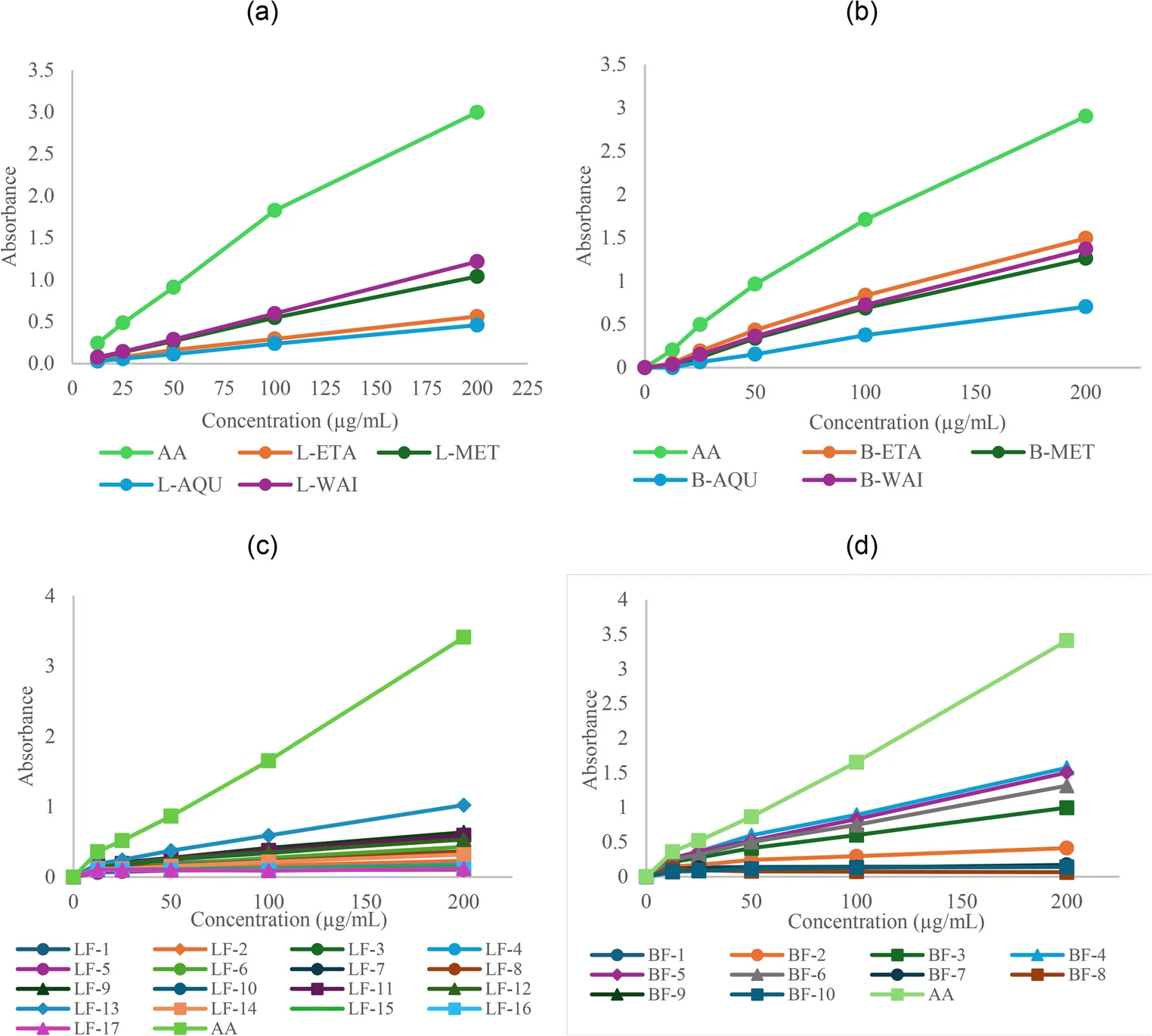
Ferric reducing activity of the extracts and fractions at different concentrations compared to the standard L-ascorbic acid (AA). Figure (**a**) represents values for leaf extracts, (**b**) represents bark extracts, (**c**) represents leaf active fractions, and (**d**) represents bark active fractions. Absorbance on the y-axis is directly proportional to the ferric reducing power of the samples. LF = leaf fractions, BF = bark fractions. Values are mean, *n* = 3

As part of the evaluation of antioxidant activity, the reducing capacity of the extracts and fractions from *A. dealbata* tissues was evaluated using the FRAP assay. The results demonstrated clear differences in reducing power among the extracts, reflecting variations in their antioxidant potential (Fig. 2).

Similar to DPPH assay, L-WAI and L-MET showed the highest reducing activity among the leaf extracts (Fig. 2a), and LF-13 showed significantly higher reducing power than other fractions, followed by LF-9 (Fig. 2c). For bark extracts, B-ETA showed the highest reducing activity along with significant activity from B-MET and B-WAI (Fig. 2b) and BF-4, BF-5, and BF-6 were the most active fractions (Fig. 2d).

#### Total antioxidant activity (TAA) assay

**Fig. 3:**
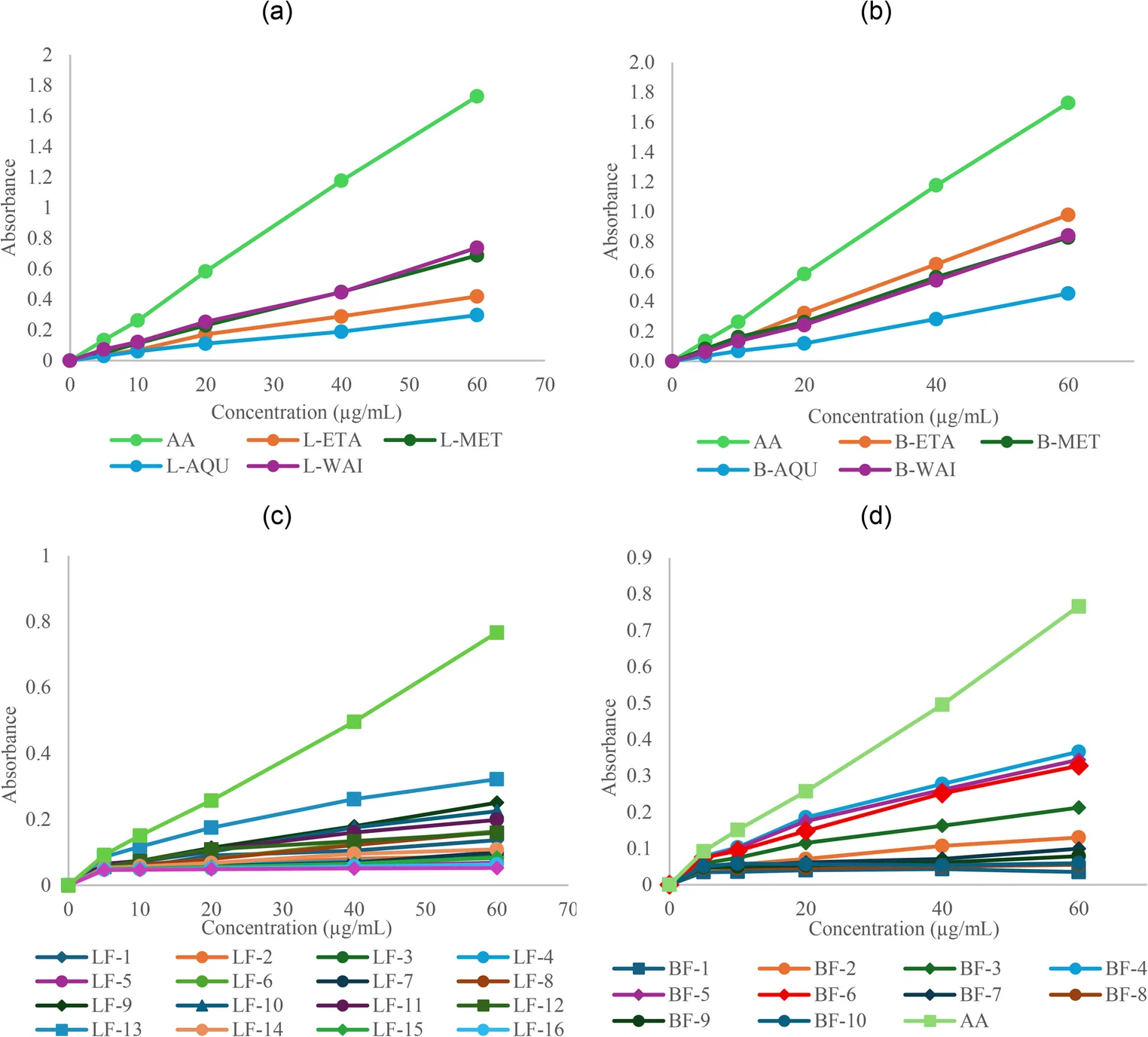
Phosphomolybdenum-based total antioxidant activity of the extracts and fractions compared to standard L-ascorbic acid (AA). Figure (**a**) represents values for leaf extracts, (**b**) represents bark extracts, (**c**) represents leaf active fractions, and (**d**) represents bark active fractions. Absorbance of the samples is directly proportional to the total antioxidant activity of the samples at each concentration. LF = leaf fractions, BF = bark fractions. Values are mean, *n* = 3

To complement the DPPH and FRAP assays, the total antioxidant activity (TAA) of the extracts was evaluated using the phosphomolybdenum method. This assay provides an overall measure of the reducing capacity of both phenolic and non-phenolic constituents, reflecting the cumulative antioxidant potential of the extracts (Prieto et al., 1999). Consistent with the DPPH and FRAP results, L-WAI and L-MET exhibited the highest TAA among the leaf extracts (Fig. 3a), while B-ETA showed the greatest activity among the bark extracts (Fig. 3b). Among the fractions, LF-13 displayed the strongest TAA in leaf fractions, followed by LF-9 (Fig. 3c), and BF-4, BF-5, and BF-6 were the most active bark fractions (Fig. 3d).

Compared to DPPH radical scavenging capability, the reducing power and total antioxidant activity were significantly lower than the standard L-ascorbic acid for all samples (Figs. 2 and 3). The differences observed between the DPPH, FRAP, and total antioxidant activity (TAA) assays likely arise from their distinct mechanisms. DPPH primarily assesses free-radical scavenging capacity through hydrogen atom and/or electron transfer mechanisms, whereas FRAP and phosphomolybdenum-based TAA assays predominantly measure electron-donating or reducing capacity (Csepregi et al., 2016; Shahidi & Samarasinghe, 2025). Variations in extract composition, including the types and structures of phenolic and flavonoid compounds, may also influence reactivity in each assay (Yu et al., 2021). Despite these mechanistic differences, the overall trend of antioxidant activity was similar in all assays demonstrating potent antioxidant activity where L-MET, B-WAI and their fractions LF-9, LF-13, BF-4, BF-5, BF-6 are the most bioactive samples.

Between the leaf and bark tissues, bark extracts showed more potent antioxidant activity than leaf extracts. This finding is consistent with the TPC and TFC content of the leaf and bark tissues, therefore the phenolic and flavonoid compounds may be responsible for the antioxidant activity of the extracts. The findings also align with traditional uses of *A. dealbata* bark for therapeutic purposes by Aboriginal Australians (Foreman & Walsh, 1993; Zola & Gott, 1992). Previous studies with the leaf, bark and flower extracts of *A. dealbata* also reported potent antioxidant activity by similar assays (Borges et al., 2020; Paula et al., 2022). These data show that *A. dealbata* could be a source of potent bioactive compounds, and may be useful for oxidative diseases, inflammation and chronic diseases caused by oxidation (Pham-Huy et al., 2008).

### Antimicrobial activity assay

**Fig. 4:**
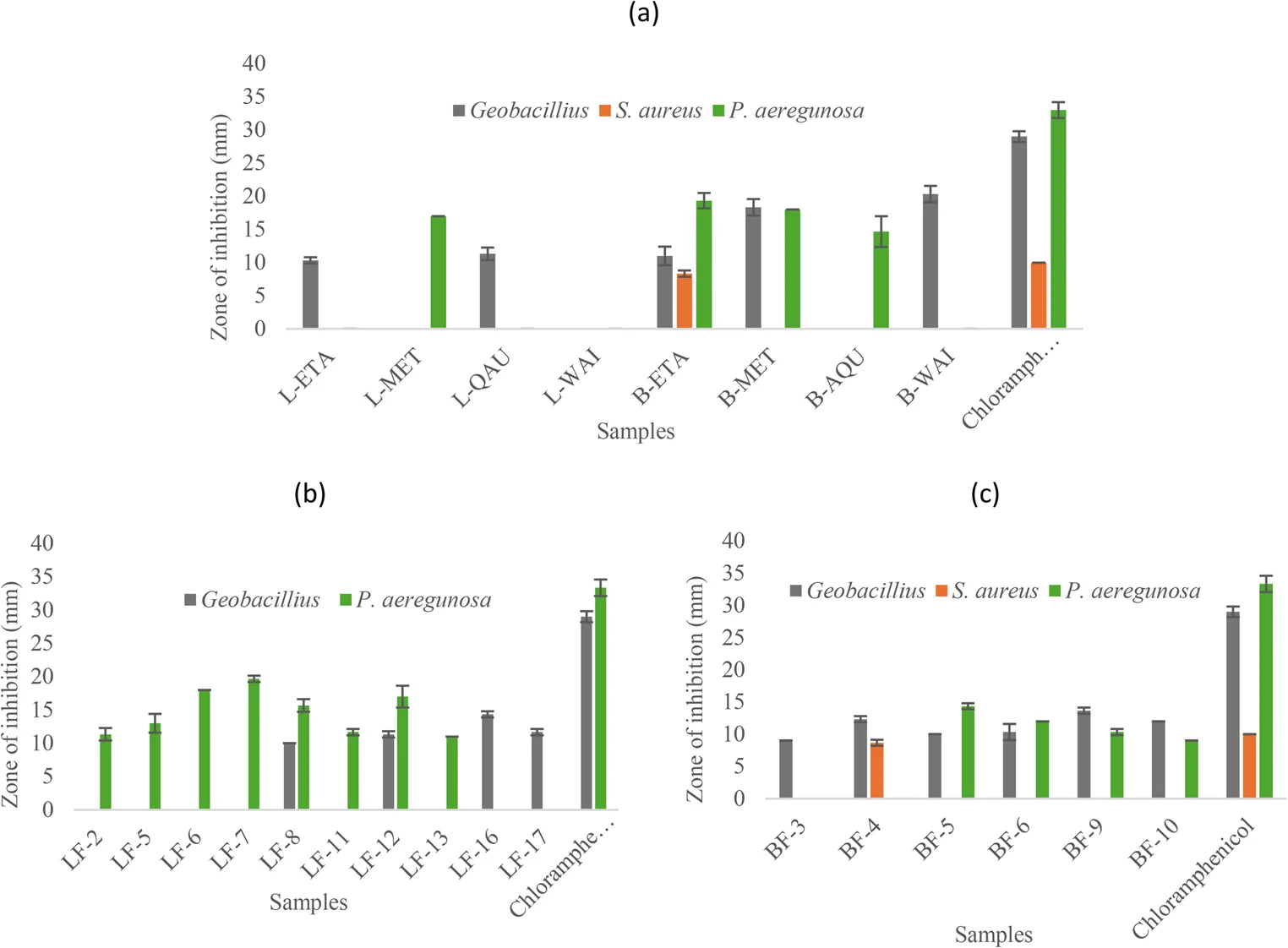
Antimicrobial activity of the extracts and fractions in disc diffusion assay, measured as diameter of the zone of inhibition (mm). Here, samples showing no inhibition against any of the tested microorganisms were excluded from the graph. Values are mean, *n* = 3

The antimicrobial screening of crude extracts showed clear differences between leaf and bark samples. Leaf extracts exhibited limited activity, with L-ETA and L-AQU showing weak inhibition only against *Geobacillius*, while L-MET demonstrated notable activity against *P. aeruginosa*. In contrast, bark extracts displayed broader antimicrobial effects; B-ETA inhibited *Geobacillius*, *S. aureus*, and *P. aeruginosa*, whereas other bark extracts showed selective activity mainly against *Geobacillius* and *P. aeruginosa*. None of the extracts showed activity against *E. coli*, likely due to the protective outer membrane typical of many Gram-negative bacteria (Hossain et al., 2022). Compared with the reference antibiotic chloramphenicol, the plant extracts showed moderate potency but still measurable antibacterial activity, in particular against Gram positive organisms (Fig. 4 and Supplementary Tables 3–4).

Based on the bioassay results, L-MET and B-ETA were selected for further fractionation to identify active constituents. Fractionation of L-MET yielded several active fractions, particularly against *P. aeruginosa*, with LF-7 showing the strongest inhibition (20 mm) followed by LF-12 (17 mm), while a smaller number of fractions were active against *Geobacillius*. This suggests that the antimicrobial compounds in the leaf methanol extract were distributed across multiple fractions but showed greater selectivity toward *P. aeruginosa*.

Similarly, fractionation of B-ETA yielded several bioactive fractions, with most showing activity against *Geobacillius* and a smaller subset inhibiting *P. aeruginosa*, while activity against *S. aureus* was limited to only one fraction (BF-4). The appearance of activity in multiple fractions suggests that the bark extract contains several antibacterial metabolites that may act independently or synergistically. These findings highlight fractions such as LF-7, LF-8, LF-12 and selected bark fractions (BF-4, BF-5, BF-6, BF-9, and BF-10) as promising candidates for further chemical characterization and isolation of potential antimicrobial compounds. However, the current study only used disc diffusion assay for testing the antimicrobial activity, which provides preliminary evidence of antimicrobial potential. Further studies using minimum inhibitory concentration (MIC) and minimum bactericidal concentration (MBC) assays are required to quantify antimicrobial potency and determine bactericidal effects.

The observed differences in antimicrobial activity may be attributed to variations in the distribution and concentration of secondary metabolites between plant tissues and extraction solvents. Bark is often richer in defensive phytochemicals such as phenolics and flavonoids, which may explain its broader antimicrobial activity compared with leaf extracts (Nisca & Tanase, 2025). This observation is also consistent with the traditional use of bark preparations for treating infectious diseases, particularly skin infections, suggesting the presence of bioactive antimicrobial constituents (Foreman & Walsh, 1993; Zola & Gott, 1992). Enhanced activity in specific fractions further indicates that chromatographic fractionation concentrated potent antimicrobial compounds, and similar selective activity has been reported in other plant species, highlighting the role of solvent polarity and compound synergy in determining antimicrobial efficacy (Khan et al., 2016; Manyawi et al., 2023).

Previous studies on crude extracts also confirmed antibacterial activity of *A. dealbata* leaf and bark extracts, especially against Gram positive organisms, while the activity was limited for Gram negative organisms (Neiva et al., 2020; Silva et al., 2016). The selective antimicrobial activity observed for *A. dealbata* extracts may be associated with the high abundance of polyphenolic compounds and the relatively low presence of lipophilic constituents in the extracts. Although polyphenols are known to exert antimicrobial effects through mechanisms such as enzyme inhibition, membrane disruption, and metal ion chelation (Daglia, 2012), lipophilic compounds generally enhance antimicrobial efficacy by facilitating penetration across bacterial cell membranes (Nadeb et al., 2025). This effect is particularly important for Gram-negative bacteria, whose outer membrane acts as a permeability barrier (Nikaido, 1989). Therefore, the limited presence of lipophilic compounds in the extracts may partly explain the selective antimicrobial activity observed.

### LC-MS based chemical characterization of the extracts

**Fig. 5:**
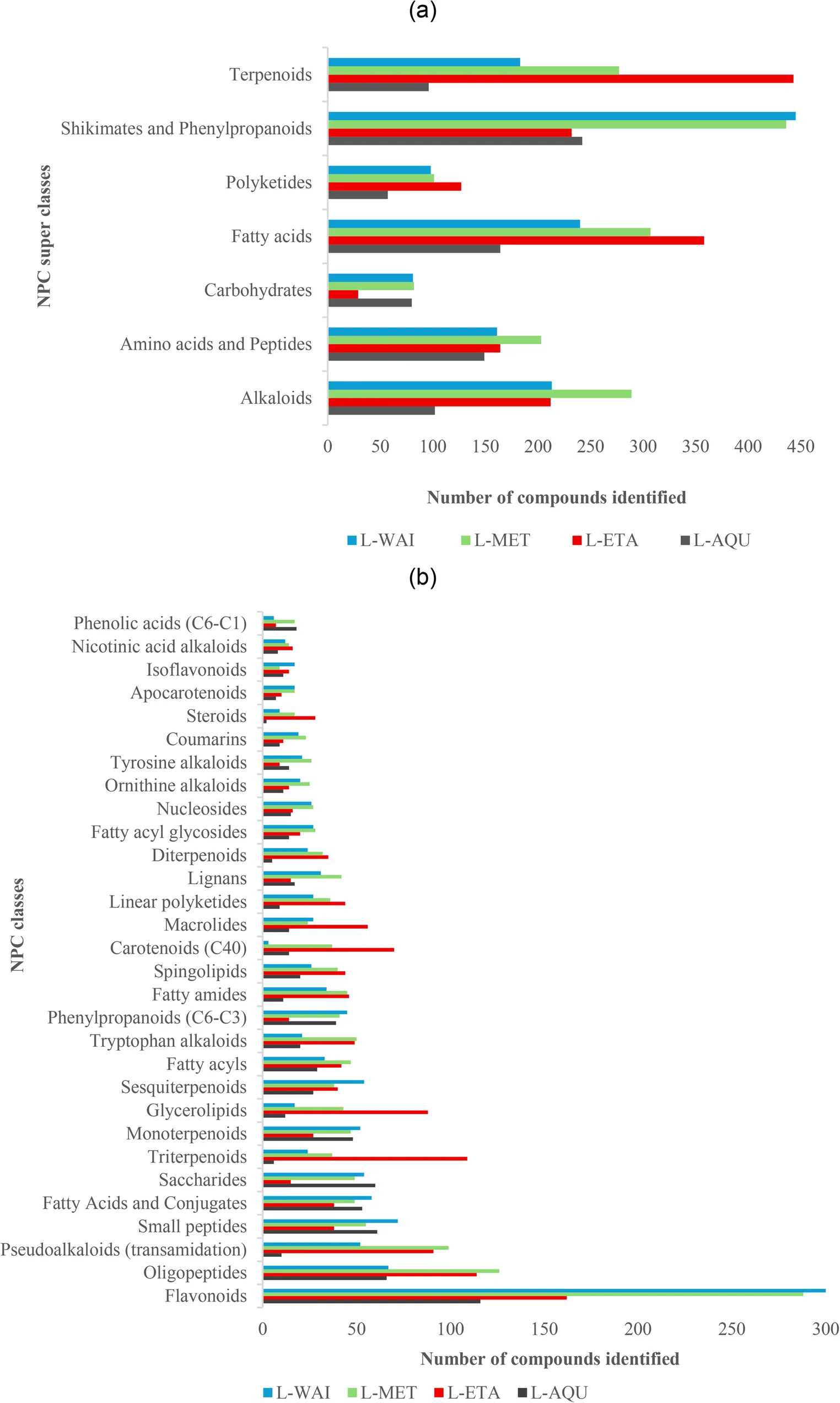
NPC super classes (**a**) and top 30 compound classes (**b**) based on in silico identification in leaf extracts (based on the number of features identified in each class). The x-axis indicates the total number of features identified in each sample. Values are average, *n* = 3

LC–MS profiling, combined with taxonomically informed metabolite annotation (TIMA), revealed a diverse metabolite composition across the four-leaf extracts (Fig. 5 and Supplementary Fig. 7). TIMA integrates chemical features from MS/MS data with biological and taxonomic information, refining metabolite annotations while providing insight into the likely bioactivity of compounds across taxa (Rutz et al., 2019). At the level of NPC (natural product classification) superclasses (Fig. 5a), shikimates and phenylpropanoids as well as terpenoids were the most abundant. L-WAI and L-MET contained the highest numbers of shikimates and phenylpropanoids (over 400 molecular features), whereas terpenoids were primarily enriched in L-ETA (over 400 molecular features). L-ETA was also rich in fatty acids, while alkaloids and amino acids were most prominent in L-MET.

Figure 5b provides a more detailed view of the NPC classes, showing flavonoids as the largest subclass of shikimates and phenylpropanoids, and the most abundant compounds across all extracts. L-WAI and L-MET exhibited the greatest flavonoid diversity. L-ETA, enriched in lipophilic terpenoids, terpenes, glycerolipids, carotenoids, and steroids, contrasts with the polar metabolite-rich L-MET and L-WAI, which also contained higher levels of sugars, peptides, and alkaloids.

The distribution of metabolites correlates with biological activity: the polar metabolite-rich extracts L-MET and L-WAI exhibited the strongest antioxidant activity and notable antimicrobial effects. These findings highlight how solvent polarity and compound polarity determine metabolite extraction and bioactivity, and illustrate that flavonoids, as major shikimate and phenylpropanoid compounds, likely contribute to both antioxidant and antimicrobial effects. The findings for lipophilic compounds are consistent with previous studies that screened *A. dealbata* leaves for phytochemical composition, which reported triterpenes and triterpenoids as the major constituents (Aguilera et al., 2015; Correia et al., 2022; Oliveira et al., 2020; Pereira et al., 1996; Perriot et al., 2010; Rodrigues et al., 2021).

However, those studies relied on GC-MS for their characterization, a technique well-suited for volatile and non-polar compounds (Tian et al., 2023), but generally inadequate for detecting polar and thermally labile metabolites such as polyphenols, which often require derivatization for GC analysis (Viñas & Campillo, 2019). In contrast, the LC–MS-based approach used in the present study allowed detection of both polar and non-polar metabolites, revealing that both polyphenols (particularly flavonoids), and triterpenes and triterpenoids are the predominant chemical constituents in *A. dealbata* leaves. The use of TIMA, together with complementary metabolomics tools such as MZmine, GNPS, and SIRIUS, enabled a more comprehensive and structurally informed characterization of the extracts. This integrated workflow not only facilitated the detection of a significantly wider range of metabolites but also improved annotation confidence through taxonomic context and enhanced spectral interpretation. Overall, this study provides a broader and more detailed view of the chemical constituents of *A. dealbata*.

#### Characterization of bark extracts

**Fig. 6:**
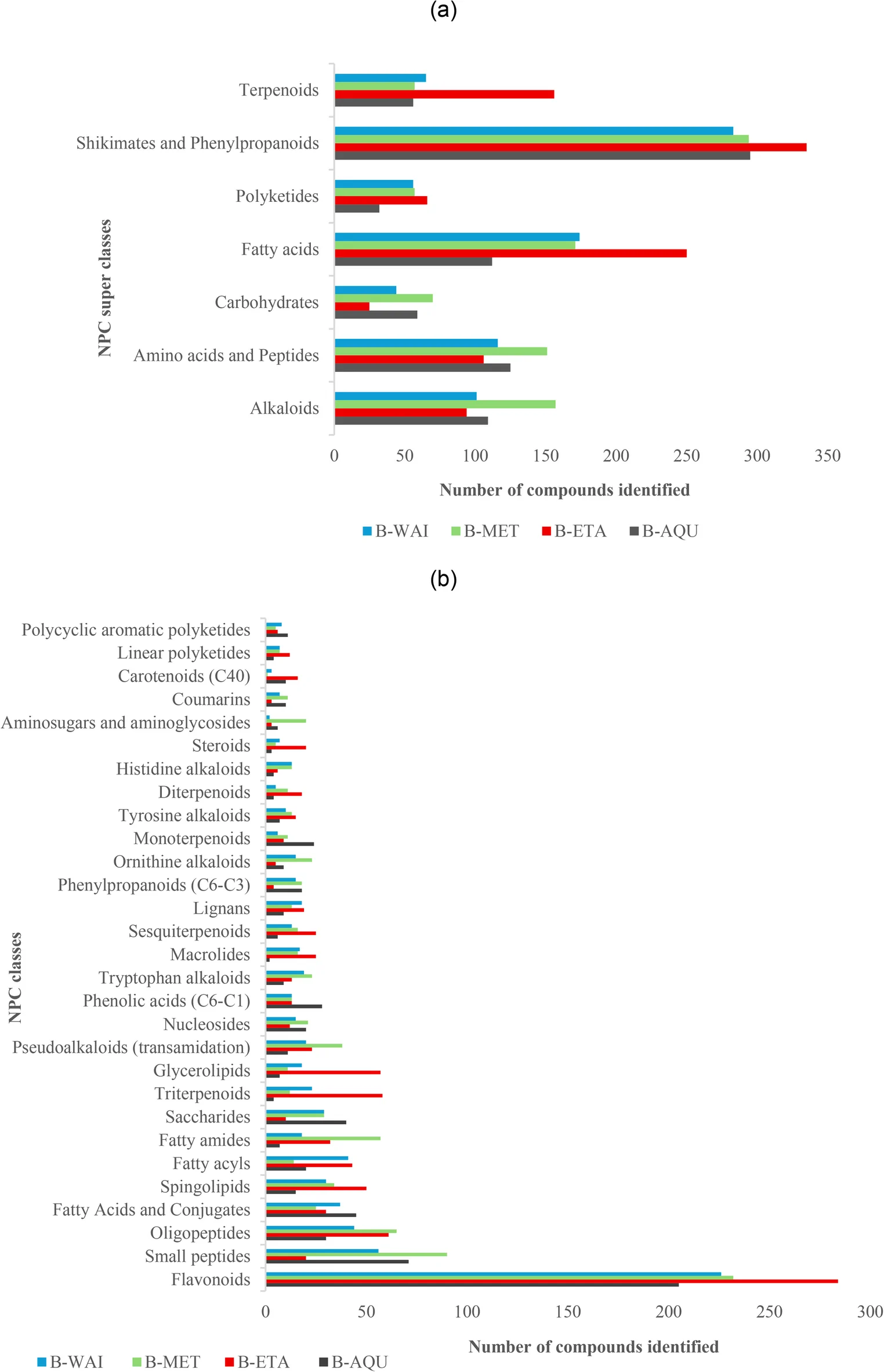
NPC super classes (**a**) and top 30 compound classes (**b**) based on in silico identification in bark extracts (based on the number of compounds identified in each class). Here, the number on the X axis indicates the total number of compounds identified in each sample. Values are average, *n* = 3

Similar to leaf extracts, LC–MS profiling of the bark extracts by TIMA revealed a rich and diverse metabolite composition (Fig. 6 and Supplementary Fig. 8). At the level of NPC superclasses (Fig. 6a), shikimates and phenylpropanoids were the most abundant across all bark samples, with B-ETA containing the highest number of compounds in this category. B-ETA was also enriched in terpenoids, polyketides, and fatty acids, whereas amino acids and alkaloids were most prominent in B-MET, indicating a differential distribution of metabolites according to both plant part and solvent polarity.

Analysis at the NPC class level (Fig. 6b) highlighted flavonoids as the dominant compounds across all bark extracts, with B-ETA exhibiting the greatest diversity. This extract also contained the highest numbers of triterpenoids and glycerolipids. These patterns reflect the influence of solvent polarity on extraction where ethyl acetate favoured lipophilic metabolites such as terpenoids, triterpenoids, and glycerolipids, while methanol extracted more polar compounds including flavonoids, amino acids, and alkaloids. The chemical profiles correlate directly with the superior bioactivity of B-ETA over other extracts, and likely the flavonoids to be the highest contributor for bioactivity.

Previously only one study reported the presence of flavonoids and glycosides from dichloromethane extract of *A. dealbata* bark using GC-MS (Freire et al., 2007), so only limited information is available on the comprehensive polyphenolic profile of the bark. The current findings using LC-MS based characterization provide new and important insight into the phytochemical composition with the identification of polyphenols like flavonoids, and flavonoid glycosides as the major constituents in *A. dealbata* bark. Molecular networking using GNPS further facilitated the characterization of the extracts, enabled comparison of the relative abundances of specific compounds across different extracts and assisted in the putative identification of potential bioactive compounds.

### Feature based molecular networking (FBMN) in GNPS

#### FBMN of the leaf extracts

**Fig. 7:**
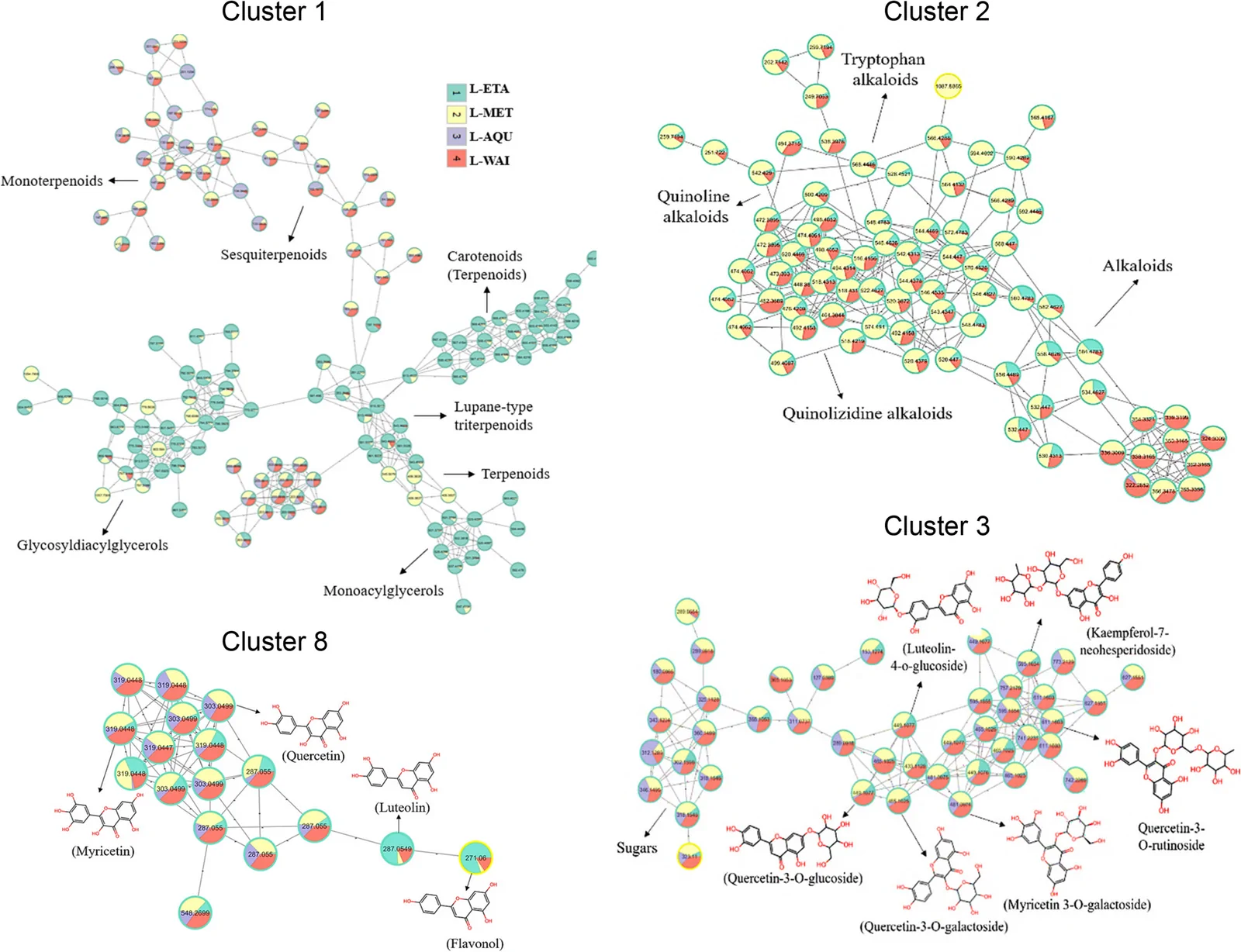
Selected clusters from the molecular network of the leaf extracts generated from positive LC–MS/MS data using GNPS2 and annotated with GNPS library and SIRIUS. Compound names and structures are based on GNPS spectral library matches. For nodes lacking direct spectral matches, SIRIUS-based formula prediction and compound class annotation were used to infer the predominant chemical classes represented within each cluster. Pie charts in the nodes represent the relative abundance based on peak area of each compound across the extracts

Feature-based molecular networking (FBMN) is a computational approach that organises LC–MS/MS features into a network based on the similarity of their MS/MS fragmentation spectra. Characterization of the extracts using metabolomic tools revealed that, for both *A. dealbata* leaf and bark extracts, positive ionization yielded a more diverse metabolite profile (Supplementary Fig. 7–8), therefore, the molecular network was generated using LC-MS/MS data acquired in positive ionization mode and the complete network is presented in Supplementary Fig. 9(a), whereas enlarged views of selected clusters are presented in Fig. 7. Clusters or individual nodes were primarily identified (tentatively) by GNPS based library matching, and SIRIUS based compound class prediction where GNPS library matching was not available. The molecular network for leaf extracts (L-ETA, L-MET, L-AQU, and L-WAI) comprised approximately twelve major clusters, reflecting the chemical diversity of the leaf metabolome and the differential extraction efficiency of the solvents employed.

Cluster 1 was primarily composed of terpenoid metabolites, including lupane-type triterpenoids and glycosyl diacylglycerols. These compounds were predominantly detected in the L-ETA extract. In contrast, monoterpenoids and sesquiterpenoids located at the upper region of the cluster were more evenly distributed among L-MET, L-AQU and L-WAI. These findings align with the previous studies on *A. dealbata*, where some studies have reported terpenes and terpenoids, particularly lupane-type triterpenoids in leaf extracts indicating consistency with previously reported findings (Aguilera et al., 2015; Oliveira et al., 2020; Rodrigues et al., 2021).

Cluster 2 was characterized by alkaloid-type metabolites, including quinoline, quinolizidine and tryptophan-derived alkaloids. This cluster was dominated by the L-MET extract, followed by L-WAI, with minimal presence in L-ETA and no significant contribution from L-AQU, reflecting the preferential extraction of nitrogen-containing metabolites in polar organic solvents. However, alkaloids have been rarely reported in the leaves of *A. dealbata*. Therefore, targeted isolation of these alkaloids, particularly from methanolic extracts, and subsequent evaluation of their biological activity may provide new insights into the bioactive potential of *A. dealbata*.

Cluster 3 represents a flavonoid glycoside-rich network from L-MET and L-WAI, containing metabolites with known bioactive properties. GNPS annotation enabled the identification of compounds from several major nodes (Supplementary Table 5), including quercetin-3-O-glucoside (465.1025 *m/z*, [M + H]^+^), myricetin-3-O-galactoside (481.0974 *m/z*, [M + H]^+^), kaempferol-3-O-glucoside (449.1076 *m/z*, [M + H]^+^), and rutin (611.1603 *m/z*, [M + H]^+^). The clustering pattern, together with shared fragment ions, supported their structural relatedness as glycosylated flavonols and flavones. The strong representation of these compounds in L-MET and L-WAI is consistent with the high polarity of glycosylated flavonoids and their known contribution to antioxidant activity (Xiao et al., 2021). Both extracts also contained some condensed tannins including procyanidin B2 (579.1490 *m/z*, [M + H]^+^) and proanthocyanins.

Cluster 8 formed another bioactivity-associated cluster, containing predominantly flavonoid aglycones. Major annotated nodes included kaempferol (287.0549 *m/z*, [M + H]^+^), myricetin (319.0450 *m/z*, [M + H]^+^) and quercetin (303.0499 *m/z*, [M + H]^+^). These metabolites were predominantly present in the L-MET, L-AQU and L-WAI extracts, further supporting the enrichment of phenolic constituents in polar extracts.

Overall, the molecular networking analysis revealed a clear solvent-dependent distribution of secondary metabolites. Non-polar terpenoids were enriched in L-ETA, whereas polar phenolics and alkaloids were concentrated in L-MET and L-WAI. Notably, the extracts that demonstrated the strongest antioxidant and moderate antimicrobial activities in bioassays, L-MET and L-WAI were characterized by a high abundance of flavonoid glycosides (Cluster 3), flavonoid aglycones (Cluster 8), and alkaloids (Cluster 2). The enrichment of quercetin-, myricetin-, luteolin-, kaempferol- and isorhamnetin-based derivatives, together with lignan-type polyphenols are consistent with the antioxidant capacity observed. Thus, the FBMN-guided metabolite annotation provides a chemical rationale for the superior bioactivity of the methanol and hydro-organic leaf extracts and highlights the importance of solvent selection in targeting bioactive phytochemical classes.

#### FBMN of the bark extracts

**Fig. 8:**
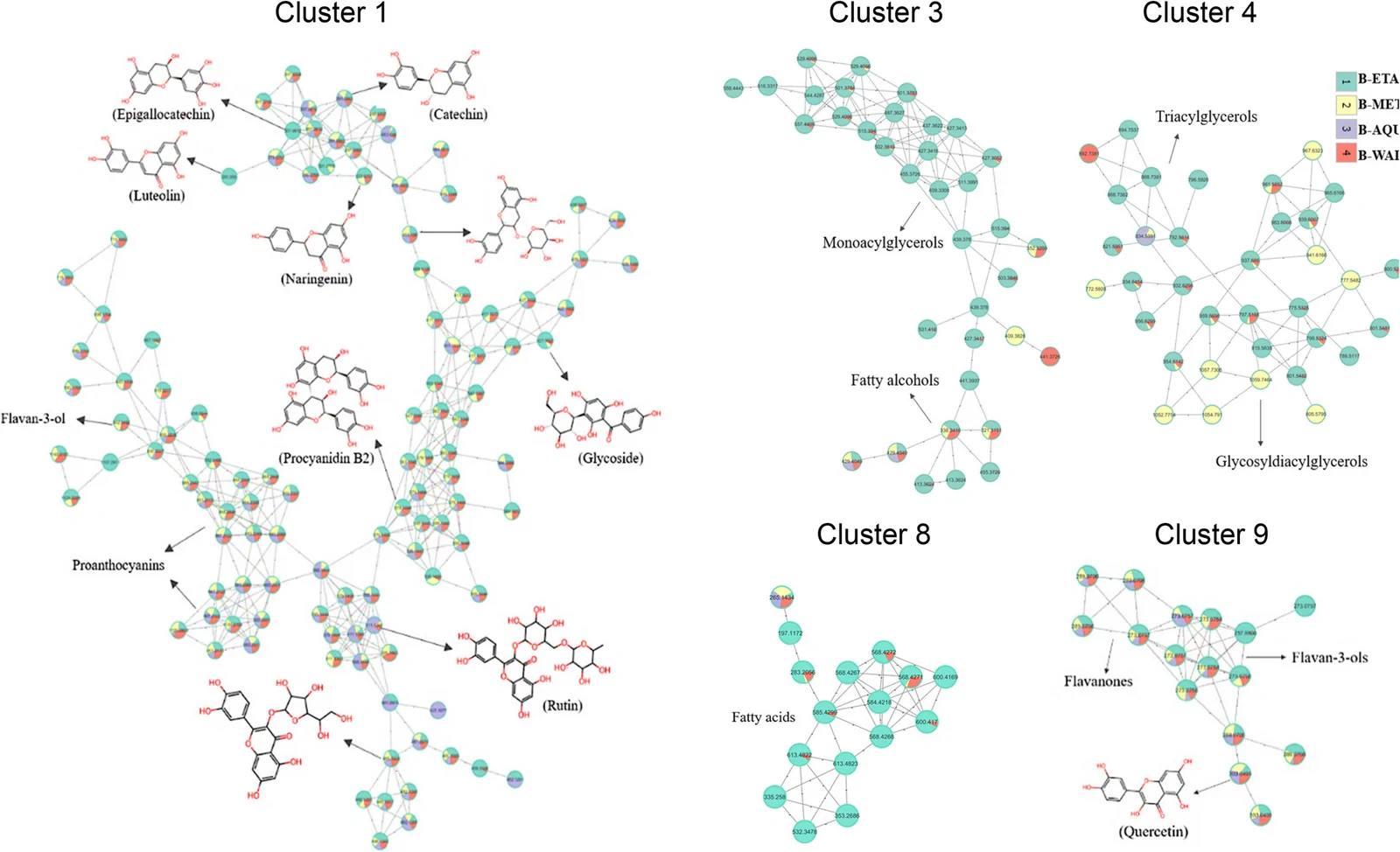
Selected clusters from the molecular network of the bark extracts generated from positive LC–MS/MS data using GNPS2 and annotated with GNPS library and SIRIUS. Compound names and structures are based on GNPS spectral library matches. For nodes lacking direct spectral matches, SIRIUS-based formula prediction and compound class annotation were used to infer the predominant chemical classes represented within each cluster. Pie charts in the nodes represent the relative abundance based on peak area of each compound across the extracts

FBMN of the bark extracts (B-ETA, B-MET, B-AQU and B-WAI) using positive mode ionization data is presented in Supplementary Fig. 9(b) which produced a comprehensive network comprising approximately 20 major clusters with solvent-dependent distribution of metabolites, which supports the observed bioactivities of the extracts. Enlarged views of selected clusters are presented in Fig. 8.

The largest cluster (Cluster 1) was dominated by flavonoids and related glycosides and represented the most chemically dense region of the network. Pie chart visualization of the relative peak areas indicated that this cluster was dominated by the ethyl acetate extract (B-ETA), with additional but comparatively lower relative amounts from B-MET and B-WAI. Library annotations identified several key flavonoids (Supplementary Table 5) including catechin (291.0860 *m/z*, [M + H]^+^), epigallocatechin (307.0812 *m/z*, [M + H]^+^), and several glycosides including rutin (611.1639 *m/z*, [M + H]^+^). SIRIUS predictions further suggested the presence of higher molecular weight flavan-3-ol oligomers and proanthocyanidins with [M + H]^+^ ions at *m/z* 563.1407, 852.2493, 835.2223 and 883.2071, and procyanidin B2 (579.1490 *m/z*, [M + H]^+^) indicating an extensive condensed tannin profile within this extract. *A. dealbata* bark is a well-known source of tannins, particularly proanthocyanidins (Abilleira et al., 2021). Given the well-established antioxidant and antimicrobial properties of flavonoids and proanthocyanidins (Rauf et al., 2019; Shahat et al., 2002), the enrichment of this cluster in B-ETA strongly explains its superior bioactivity.

Cluster 9 represented another flavonoid-rich region of the network and contained quercetin (303.045 *m/z*, [M + H]^+^), together with additional flavanones and flavan-3-ols. Like Cluster 1, this cluster was predominantly derived from B-ETA, with notable contributions from B-MET and B-WAI. The consistent enrichment of polyphenolic metabolites in B-ETA across multiple clusters further supports its chemical composition and biological potency.

In contrast, Cluster 2 was mainly composed of sugars, small peptides, and organic acids, and was dominated by the aqueous and methanolic extracts. Clusters 3 and 4 were mainly associated with B-ETA and consisted of non-polar metabolites such as glycerols and fatty alcohols. Similarly, Cluster 8 contained fatty acids and related lipophilic compounds, also dominated by B-ETA. These clusters were predominantly associated with B-ETA, consistent with the extraction of lipophilic constituents by ethyl acetate.

Overall, the molecular network suggests B-ETA as an enriched source of bioactive flavonoids and proanthocyanidins. This chemical distribution closely mirrored the bioassay results and therefore indicate that the superior antioxidant and antimicrobial activities of B-ETA may be attributed to its higher abundance of flavonoids, flavonoid glycosides and condensed tannins.

### Characterization of fractions and identification of bioactive compounds

#### Characterization of the bioactive fractions by LC-MS analysis

Based on bioassay results, chemical characterization, and molecular networking, L-MET and B-ETA exhibited the most promising profile, therefore they were selected for further fractionation and characterization.

Fractionation of L-MET using preparative HPLC yielded 17 fractions, while B-ETA yielded 10 fractions. Targeted LC–MS analysis of L-MET, B-ETA, and their fractions was performed using 11 standard polyphenols in negative ionization mode due to higher ionisation efficiency of the selected compounds in negative ionisation mode, and the results are presented in Supplementary Table 6. Among the 11 targeted compounds, isorhamnetin and genistein were not detected in any sample, while naringenin was detected in trace amounts in leaf extract and fractions. Of the remaining compounds, catechin, epigallocatechin, procyanidin B2, and rutin were the most abundant in bark extracts and their corresponding fractions.

In bark fractions, the highest level of catechin was detected in bark fraction 3 (BF-3; 144 µg/mg of sample), while procyanidin B2 was highest in BF-4 (252 µg/mg of sample). BF-5 also contained detectable amounts of procyanidin B2 (17.9 µg/mg of sample) and rutin (3.10 µg/mg of sample). These findings are consistent with previous reports describing *Acacia* bark as a rich source of flavan-3-ols and condensed tannins. For example, catechin has been reported as a major constituent of *A. catechu* heartwood, with reported levels ranging from 3.30 to 66.9% depending on extraction and analytical approaches (Kumari et al., 2022). Another study characterised *A. mearnsii* bark proanthocyanidins and showed that the bark extract contains high levels of condensed tannins (24% of dry weight) (Chen et al., 2018). Procyanidin B2, catechin, and epigallocatechin have been reported to exhibit antioxidant and other biological activities (Bae et al., 2020; Chen et al., 2023; Lei et al., 2025; Millán-Laleona et al., 2026). Their detection in the extracts and fractions is consistent with the potential contribution of polyphenols to the observed bioactivities. However, the individual contributions of these compounds were not evaluated in the present study and require further investigation.

BF-6 also exhibited strong biological activity; however, none of the targeted standards were predominant in this fraction. Instead, untargeted LC–MS/MS analysis tentatively identified proanthocyanidin-type oligomers, with characteristic [M-H]^−^ ions at *m/z* 545.1456, 561.1402, 833.2096 and 849.2043, which might contribute to its observed bioactivity (Yang et al., 2021).

In L-MET and its fractions, rutin was the dominant identified compound, with the highest abundance observed in LF-1 (19.7 µg/mg of sample) and LF-9 (57.9 µg/mg of sample). A previous study has quantified rutin in leaves of different *Acacia* species, including *A. salicina* (10.42 µg/mg sample), *A. laeta* (2.67 µg/mg sample), *A. hamulosa* (1.36 µg/mg sample), and *A. tortilis* (0.31 µg/mg sample), with *A. salicina* exhibiting the highest reported rutin content (Alam et al., 2017). Therefore, the rutin content in *A. dealbata* leaf is significantly higher than other *Acacia* species.

LF-9 was among the most bioactive leaf fractions and contained the highest rutin content (57.9 µg/mg of sample). Rutin, a flavonoid O-glycoside, is well documented for its antioxidant and anti-inflammatory properties (Al-Dhabi et al., 2015; Gullón et al., 2017). Along with rutin, myricetin-3-O-galactoside (479.0831 *m/z*, [M-H]^−^), quercetin-3-O-glucoside (463.0879 *m/z*, [M-H]^−^), and a proanthocyanin (723.5033 *m/z*, [M-H]^−^) were detected tentatively using untargeted analysis and are the major compounds in LF-9 based on relative peak areas. Another highly bioactive fraction, LF-13, contained only trace levels of rutin, however, untargeted LC–MS/MS analysis tentatively identified several phenolic glycosides as major constituents, including kaempferol-3-O-glucoside (447.0934 *m/z*, [M-H]^−^), a protocatechuic glucoside (435.1297 *m/z*, [M-H]^−^), kaempferol 7-neohesperidocide (593.1517 *m/z*, [M-H]^−^) and a proanthocyanin (723.5033 *m/z*, [M-H]^−^). Flavonoid O-glycosides are widely reported to exhibit diverse biological activities, including antioxidant, anti-inflammatory, antimicrobial, and cardioprotective effects (Kumar & Pandey, 2013; Yang et al., 2018). The presence of these compounds may be associated with the observed bioactivity of LF-13; however, further studies using authentic standards and dose–response assays are required to establish their individual contributions. Overall, this LC–MS-based targeted analysis confirmed the presence of key polyphenolic compounds in *A. dealbata* leaf and bark extracts, supporting their annotation from untargeted LC–MS/MS metabolomics.

#### Characterization of the bioactive fractions by NMR spectroscopy

The NMR profiles of the bark (BF4–BF6) and leaf (LF-13) fractions are markedly different. Owing to the complexity and extensive signal overlap present in the crude bioactive fractions, two-dimensional NMR experiments were employed to establish proton–proton and proton–carbon connectivity and facilitate class-level structural annotation. Bark fractions show dense aromatic/polyphenolic signals (predominantly δH ~ 5.9–7.1 ppm, δC ~ 95–130 ppm) with relatively little intensity in the oxygenated aliphatic/carbohydrate region. In addition, a weak but reproducible ^13^C resonance at 168–169 ppm is observed in all three bark fractions, suggesting the presence of minor esterified or carboxyl-containing constituents. Representative spin systems and characteristic chemical shift ranges for each fraction are summarised in Table 3. In contrast, LF-13 is dominated by oxygenated aliphatic networks (δH 3.0–4.1 ppm, δC 55–79 ppm) and exhibits a weak anomeric ^1^–^13^ C cross peak at 5.52 ppm/ 102.8 ppm. This trend mirrors literature reports that *A. dealbata* bark is rich in flavonoids and condensed tannins, whereas leaf extracts often contain higher proportions of glycosylated and other oxygenated metabolites (Correia et al., 2022; Uushona et al., 2021).

**Table 3:** Representative NMR spin systems and characteristic chemical shift ranges identified in the bioactive fractions of *A. dealbata*

Fraction	Key spin systems	Representative chemical shifts (δH / δC, ppm)
BF4	SS5, SS8, SS12	δH 5.94–6.85, δC 95.0–119.8
BF5	SS3, SS5, SS6	δH 5.93–6.91, δC 95.2–128.6
BF6	SS17, SS20, SS7	δH 6.21–7.12, δC 102.6–128.5 (SS7: δH 2.68–4.09, δC 67.3)
LF13	SS11, SS20 (+ anomeric)	δH 1.07–4.03, δC 61.3–77.1 (δH 5.52/δC 102.8 anomeric)

Here, the listed spin systems (SS) correspond to those discussed in the text and are derived from combined COSY and HSQC analyses using the NMRMine workflow. Chemical shifts are reported as representative ranges (δH/δC, ppm) for the major structural features contributing to the class-level metabolite annotations

In the bark fractions BF4–BF6, 2D NMR (COSY, HSQC) and spin-system analysis reveal multiple strongly coupled aromatic networks, consistent with flavonoid-type polyphenols and the characteristic C₆–C₃–C₆ flavonoid framework reported for flavan-3-ol-derived metabolites (Kulić et al., 2025). For example, BF4 contains a large aromatic spin system SS5 (δH 5.94–6.68 ppm, δC 95.0–129.8 ppm) and additional aromatic systems SS8 (δH: 6.67, 6.75; δC: 114.9, 118.6) and SS12 (δH: 6.78, 6.85; δC: 114.0, 114.7, 119.8). BF5 is dominated by a large aromatic spin system (SS3) containing resonances at δH 5.93–6.91 ppm associated with carbons at 95.2–129.8 ppm, together with several downfield resonances at δH 10.03–11.72 ppm. Additional aromatic spin systems, including SS5, SS6 and SS10, occupy similar aromatic regions (δC: 105–129 ppm), further supporting the predominance of highly substituted phenolic structures. Similarly, BF6 features SS17 (δH: 6.23–7.12; δC 104.9–128.5) and SS20 (δH: 6.21–7.11; δC: 102.6–128.5) in the aromatic region. Several of these proton-carbon correlations fall within chemical shift ranges reported for phloroglucinol- and catechol-type aromatic units commonly encountered in flavan-3-ols and proanthocyanidins (Crestini et al., 2016).

Each bark fraction also contains smaller oxygenated aliphatic spin systems with resonances typically observed between δH 3.3–4.6 ppm and δC 67–83 ppm. These chemical shifts are consistent with oxygenated carbon environments commonly encountered in flavonoid and flavonoid-derived polyphenols, although the corresponding spin systems are not sufficiently diagnostic to permit more specific structural assignments. No clear anomeric proton-carbon correlations are observed in the bark fractions. Overall, the BF4–BF6 patterns point to polyphenolic compositions, including flavonoid- and flavan-3-ol-derived constituents, rather than carbohydrate-rich metabolites.

The leaf fraction (LF-13) gives a contrasting NMR signature. Extracted spin systems SS11 and SS20 each consist entirely of oxygenated aliphatic resonances. For example, SS11 shows δH 1.07, 3.35–3.89, 4.03 ppm with δC 61.3–65.9, and SS20 shows δH 3.07–3.92 ppm with δC 61.3–77.1 ppm. These ranges (non-anomeric protons clustering at δH 3.0–4.0) are typical of sugar or glycoside backbones. Crucially, a weak HSQC cross peak at δH 5.52/δC 102.8 ppm is observed, clearly indicating an anomeric sugar proton. This correlation falls within the range reported for the anomeric resonances of flavonoid glycosides such as hyperoside, isoquercitrin, rutin and related O-glycosylated flavonoids, supporting the presence of glycosidic metabolites in LF-13 (Kulić et al., 2025). Only limited aromatic resonances are observed in LF-13. A small number of COSY-derived spin systems contain signals in the aromatic region (δH ~ 6.9–8.0 ppm), although corresponding HSQC correlations are weak and observed only at low intensity. These observations suggest the presence of minor aromatic metabolites within the fraction, but their low abundance precludes more detailed structural interpretation. A H​^1^​–^13^​C HMBC correlation from a CH₂ (δH 2.28) to a carbonyl (δC 173.45) is noted, consistent with a minor carboxylate/ester functionality. Taken together, the NMR data indicate LF-13 is dominated by oxygenated and glycosidic metabolites (e.g. sugar conjugates and other oxygenated natural products), with only limited evidence for minor aromatic metabolites.

These NMR-derived class assignments agree well with the untargeted LC-MS/MS-based annotations using GNPS and SIRIUS. For example, MS-based identifications of catechin, epigallocatechin, quercetin derivatives, flavonoid glycosides and related polyphenols in the bark fractions are consistent with the aromatic spin systems and polyphenol-rich NMR profiles present in the bark fractions. Likewise, the detection of numerous glycosylated metabolites in the leaf extract and fractions, including flavonoid glycosides and related sugar conjugates, agrees with the extensive oxygenated aliphatic networks and the weak anomeric HSQC correlation (δH 5.52/δC 102.8 ppm) present in LF-13. We use conservative terminology (e.g. “flavonoid-/flavan-3-ol-derived” and “glycosidic/carbohydrate-like”) because the NMR data support class-level rather than compound-specific assignments. Collectively, the combined COSY, HSQC and HMBC experiments, supported by NMRMine-assisted spin-system extraction, reveal a clear compositional distinction between the tissues: BF4–BF6 are enriched in tannins, flavonoids, and flavan-3-ol-derived polyphenols, whereas LF-13 is dominated by glycosidic and other oxygenated metabolites.

## Conclusion

The present study evaluated the bioactive potential of *A. dealbata* leaf and bark extracts and provided a comprehensive characterization of their chemical composition. Previous studies have reported only a limited number of constituents from these tissues, and investigations of biological activity have largely been restricted to crude extracts without clear identification of the compounds responsible for activity. Therefore, the application of advanced LC–MS-based metabolomics in this study addressed these limitations and enabled a more in-depth and structurally resolved characterization of the extracts. Integrated chemical profiling and bioactivity assessment identified the leaf methanolic extract (L-MET) and bark ethyl acetate extract (B-ETA) as the most promising extracts from leaf and bark, respectively. Untargeted metabolomics revealed that bark extracts were predominantly enriched in polyphenols, particularly flavonoids and glycosylated derivatives, while leaf extracts contained a broader chemical diversity, including polyphenols alongside terpenes, terpenoids, and alkaloids.

Further fractionation of the most bioactive extracts, combined with targeted LC–MS and NMR analysis, suggest that polyphenolic constituents, especially flavonoids, condensed tannins, and flavonoid glycosides may be associated with the observed antioxidant and antimicrobial activities. Overall, *A. dealbata* leaf and bark exhibited strong antioxidant activity and moderate antimicrobial activity, supporting their complementary bioactive profiles. These findings collectively highlight *A. dealbata* leaf and bark as valuable and chemically diverse sources of bioactive secondary metabolites.

## Supplementary Information

Supplementary Material 1.

## Data Availability

All data supporting the findings of this study are available within the paper and its Supplementary Information. Raw LC-MS files can be provided on request or GNPS job provided.
