# Supplementary material for: Metabolomics and bioassay-guided characterization identifies bioactive polyphenols in Acacia dealbata leaf and bark extracts
Source: Metabolomics. 2026 Nov 14;22(6):182. doi: 10.1007/s11306-026-02558-7 (PMC13769759; doi:10.1007/s11306-026-02558-7)
Supplement: Supplementary file 1 [file 11306_2026_2558_MOESM1_ESM.docx]

**Metabolomics and bioassay-guided characterization identifies bioactive polyphenols in *Acacia dealbata* leaf and bark extracts**

**Journal: Metabolomics**

Md Sanowar Hossain, Hemavaruni N. Fernando, Madhuri Hande, Jacqui L. Adcock, Damien L. Callahan*****

***Corresponding author**: Damien L. Callahan

Contact: School of Life and Environmental Sciences

Deakin University

221 Burwood Highway, Burwood, Victoria 3125, Australia

Mobile No: +61 437247166

Email: [damien.callahan@deakin.edu.au](mailto:damien.callahan@deakin.edu.au)

**Supplementary data**

**Measurement of total phenolic and flavonoid content**

(a)

| Concentration of gallic acid (µg/mL) | Absorbance at 760 nm |
| --- | --- |
| 20 | 0.248 |
| 40 | 0.681433 |
| 60 | 1.07257 |
| 80 | 1.44347 |
| 100 | 1.80623 |

(b)

| Concentration of quercetin (µg/mL) | Absorbance at 506 nm |
| --- | --- |
| 20 | 0.0494333 |
| 40 | 0.0875667 |
| 60 | 0.126667 |
| 80 | 0.1756 |
| 100 | 0.217833 |
| 200 | 0.397533 |

**Supplementary Fig. 1** Standard gallic acid (a) and quercetin (b) curve for quantitative measurement of total phenolic and flavonoid content respectively in leaf and bark extracts.

**Supplementary Table 1**: Total phenolic and flavonoid content of the leaf and bark extracts.

| **Sample** | **Mass of  extract (µg)** | **Total phenolic content** | | **Total flavonoid content** | |
| --- | --- | --- | --- | --- | --- |
|  |  | **Average absorbance (AU)** | **GA equivalent Concentration (mg/g)** | **Average absorbance**  **(AU)** | **QCT equivalent Concentration (mg/g)** |
| L-ETA | 250 | 0.998 | 229.2±3.1 | 0.075 | 129.6±9.2 |
| L-MET | 250 | 1.786 | 391.5±4.6 | 0.114 | 211.2±14.4 |
| L-AQU | 250 | 0.699 | 167.5±2.9 | 0.036 | 46.5±2.4 |
| L-WAI | 250 | 1.956 | 426.7±1.7 | 0.115 | 213.9±24.4 |
| B-ETA | 250 | 2.639 | 567.5±2.5 | 0.220 | 434.0±8.6 |
| B-MET | 250 | 2.180 | 472.9±8.2 | 0.155 | 311.1±28.6 |
| B-AQU | 250 | 1.477 | 327.9±1.8 | 0.103 | 188.1±3.1 |
| B-WAI | 250 | 2.273 | 491.9±3.9 | 0.169 | 326.7±13.4 |

Values are mean ± SD, n =3, measured on the dry weight basis of samples. Total phenolic content (TPC) was expressed as gallic acid (GA) equivalent while the total flavonoid content was expressed as quercetin (QCT) equivalent using standard curves. AU = Absorbance unit.

**Antioxidant activity of the extracts and fractions**

**DPPH radical scavenging assay**

(a)

% Scavenging

| Concentration (µg/mL) | AA | L-ETA | L-MET | L-AQU | L-WAI |
| --- | --- | --- | --- | --- | --- |
| 0 | 0 | 0 | 0 | 0 | 0 |
| 5 | 47.8851 | 14.5712 | 39.0312 | 14.2866 | 34.0706 |
| 10 | 96.5981 | 30.7916 | 70.5342 | 23.1665 | 67.2487 |
| 20 | 96.9797 | 61.0076 | 96.3782 | 39.6521 | 96.3265 |
| 40 | 96.9991 | 93.3773 | 96.4429 | 69.6676 | 96.4946 |
| 60 | 97.0056 | 92.2778 | 96.4817 | 92.1097 | 96.5334 |

(b)

% Scavenging

| Concentration (µg/mL) | LF-1 | LF-2 | LF-3 | LF-4 | LF-5 | LF-6 | LF-7 | LF-8 | LF-9 | LF-10 | LF-11 | LF-12 | LF-13 | LF-14 | LF-15 | AA |
| --- | --- | --- | --- | --- | --- | --- | --- | --- | --- | --- | --- | --- | --- | --- | --- | --- |
| 0 | 0 | 0 | 0 | 0 | 0 | 0 | 0 | 0 | 0 | 0 | 0 | 0 | 0 | 0 | 0 | 0 |
| 5 | 29.0248 | 6.27603 | 2.48456 | 6.3622 | 5.70157 | 10.2111 | 5.47178 | 17.4781 | 32.2418 | 32.644 | 31.653 | 35.387 | 53.3965 | 11.4319 | 0.0718081 | 46.7758 |
| 10 | 35.2 | 14.2 | 6.6 | 9.2 | 37.0817 | 69.9698 | 41.9216 | 61.1805 | 71.6789 | 69.1369 | 65.331 | 70.573 | 79.506 | 14.0313 | 0.1 | 80.2384 |
| 20 | 41.0742 | 18.9573 | 10.6132 | 16.5159 | 44.6 | 76.2 | 53 | 70.1 | 82.0049 | 82.7373 | 78.7448 | 78.0698 | 79.032 | 32.3711 | 9.11963 | 80.7123 |
| 40 | 46.7 | 22.6196 | 12.2936 | 25.2765 | 47.3 | 77.4379 | 61.4 | 78.4145 | 82 | 83 | 78.8453 | 78.8597 | 80 | 63.1768 | 21.7148 | 81.1863 |
| 60 | 61.3529 | 35.3152 | 23.8259 | 36.9381 | 49.2 | 77.5 | 74.1 | 75.9299 | 82 | 83 | 78.8 | 79 | 81.1 | 74.9246 | 24.86 | 81.1001 |

(c)

% Scavenging

| Concentration (µg/mL) | AA | B-ETA | B-MET | B-AQU | B-WAI |
| --- | --- | --- | --- | --- | --- |
| 0 | 0 | 0 | 0 | 0 | 0 |
| 5 | 52.5446 | 45.9414 | 32.7484 | 21.7784 | 44.8323 |
| 10 | 95.3913 | 86.6801 | 69.5316 | 38.433 | 79.2346 |
| 20 | 96.6945 | 94.9792 | 96.0864 | 72.3566 | 94.0322 |
| 40 | 97.0609 | 96.8335 | 96.162 | 95.41 | 95.4242 |
| 60 | 97.0888 | 97.0951 | 96.2821 | 95.7065 | 96.0329 |

(d)

% Scavenging

| Concentration (µg/mL) | BF-1 | BF-2 | BF-3 | BF-4 | BF-5 | BF-6 | BF-7 | BF-8 | BF-9 | BF-10 | AA |
| --- | --- | --- | --- | --- | --- | --- | --- | --- | --- | --- | --- |
| 0 | 0 | 0 | 0 | 0 | 0 | 0 | 0 | 0 | 0 | 0 | 0 |
| 5 | 2.07736 | 10.2674 | 29.9069 | 58.011 | 53.4265 | 53.713 | 13.0253 | 6.44699 | 12.0821 | 11.2942 | 60.8047 |
| 10 | 11.127 | 32.5454 | 72.8988 | 81.6141 | 81.7216 | 79.7039 | 29.8114 | 9.62273 | 19.3052 | 19.3171 | 82.5215 |
| 20 | 16.2846 | 53.6175 | 79.7159 | 81.3395 | 81.638 | 81.3515 | 49.0091 | 12.4761 | 26.5759 | 26.2178 | 83.2856 |
| 40 | 23.042 | 80.4561 | 81.0172 | 81.4351 | 81.7096 | 81.1963 | 75.788 | 14.5654 | 26.755 | 28.7249 | 83.5244 |
| 60 | 29.5965 | 80.5396 | 81.1485 | 81.9126 | 81.9365 | 81.829 | 81.65 | 16.8577 | 30.6113 | 30.3964 | 83.6437 |

**Supplementary Fig. 2** DPPH radical scavenging activity of the leaf extracts (a), leaf methanolic fractions (b), bark extracts (c), and bark ethyl acetate fractions (d). AA = L-ascorbic acid, LF = leaf fraction (from L-MET), BF= bark fraction (from B-ETA).

**Ferric reducing power assay**

(a)

| Concentration (µg/mL) | Absorbance |
| --- | --- |
| 12.5 | 0.242733 |
| 25 | 0.484267 |
| 50 | 0.910567 |
| 100 | 1.82537 |

(b)

| Concentration (µg/mL) | Absorbance |
| --- | --- |
| 12.5 | 0.2037 |
| 25 | 0.497833 |
| 50 | 0.964433 |
| 100 | 1.71027 |

**Supplementary Fig. 3** Standard L-ascorbic acid curve for measuring reducing power activity of *A. dealbata* leaf (a) and bark (b) extracts.

Absorbance

| Concentration (µg/mL) | Absorbance |
| --- | --- |
| 12.5 | 0.361133 |
| 25 | 0.5183 |
| 50 | 0.8655 |
| 100 | 1.6534 |
| 200 | 3.40907 |

**Supplementary Fig. 4** Standard L-ascorbic acid curve for measuring ferric reducing power activity of *A. dealbata* leaf and bark fractions.

**Total antioxidant activity assay**

| Concentration (µg/mL) | Absorbance |
| --- | --- |
| 5 | 0.1352 |
| 10 | 0.262967 |
| 20 | 0.583767 |
| 40 | 1.17693 |
| 60 | 1.7294 |

**Supplementary Fig. 5** Standard L-ascorbic acid curve for measuring total antioxidant activity of *A. dealbata* leaf and bark extracts.

Absorbance

| Concentration (µg/mL) | Absorbance |
| --- | --- |
| 5 | 0.0923333 |
| 10 | 0.151167 |
| 20 | 0.2574 |
| 40 | 0.4961 |
| 60 | 0.766867 |

**Supplementary Fig. 6** Standard L-ascorbic acid curve for measuring total antioxidant activity of *A. dealbata* leaf and bark fractions.

**Fractionation of L-MET and B-ETA**

**Supplementary Table 2:** Quantity of the fractions obtained from L-MET and B-ETA extracts after separation by preparative HPLC.

| **Leaf fractions** | **Quantity (mg)** |  | **Bark fractions** | **Quantity (mg)** |
| --- | --- | --- | --- | --- |
| LF-1 | 2.5 |  | BF-1 | 1.0 |
| LF-2 | 1.0 |  | BF-2 | 1.2 |
| LF-3 | 3.2 |  | BF-3 | 2.5 |
| LF-4 | 2.2 |  | BF-4 | 11.1 |
| LF-5 | 13.4 |  | BF-5 | 21.0 |
| LF-6 | 2.5 |  | BF-6 | 17.3 |
| LF-7 | 1.3 |  | BF-7 | 4.0 |
| LF-8 | 2.8 |  | BF-8 | 1.7 |
| LF-9 | 6.1 |  | BF-9 | 1.2 |
| LF-10 | 1.7 |  | BF-10 | 1.0 |
| LF-11 | 1.0 |  |  | |
| LF-12 | 1.0 |  |  |  |
| LF-13 | 10.4 |  |  |  |
| LF-14 | 6.3 |  |  |  |
| LF-15 | 1.8 |  |  |  |
| LF-16 | 2.1 |  |  |  |
| LF-17 | 1.1 |  |  |  |

Here, LF = leaf fraction, BF = bark fraction, numbers indicate the respective fraction numbers. Yield was calculated on dry weight (mg) basis.

**Assay of the antimicrobial activity**

**Supplementary Table 3:** Antimicrobial activity of the leaf and bark extracts of *A. dealbata*.

| **Samples** | ***Geobacillius*** | ***S. aureus*** | ***P. aeruginosa*** |
| --- | --- | --- | --- |
|  | Zone of inhibition (mm) | | |
| L-ETA | 10.3±0.5 | NA | NA |
| L-MET | NA | NA | 17.0±0.0 |
| L-QAU | 11.3±0.9 | NA | NA |
| L-WAI | NA | NA | NA |
| B-ETA | 11.0±1.4 | 8.3±0.5 | 19.3±1.2 |
| B-MET | 18.3±1.2 | NA | 18.0±0.0 |
| B-AQU | NA | NA | 14.7±2.3 |
| B-WAI | 20.3±1.2 | NA | NA |
| Chloramphenicol | 29.0±0.8 | 10.0±0.0 | 33.3±1.2 |

**Supplementary Table 4:** Antimicrobial activity of the leaf and bark fractions of *A. dealbata*.

| **Samples** | ***Geobacillius*** | ***P. aeruginosa*** | **Samples** | ***Geobacillius*** | ***S. aureus*** | ***P. aeruginosa*** |
| --- | --- | --- | --- | --- | --- | --- |
|  | Zone of inhibition (mm) | |  | Zone of inhibition (mm) | | |
| LF-2 | NA | 11.3±0.9 | BF-3 | 9.0±0.0 | NA | NA |
| LF-5 | NA | 13.0±1.4 | BF-4 | 12.3±0.5 | 8.7±0.5 | NA |
| LF-6 | NA | 18.0±0.0 | BF-5 | 10.0±0.0 | NA | 14.3±0.5 |
| LF-7 | NA | 19.7±0.5 | BF-6 | 10.3±1.2 | NA | 12.0±0.0 |
| LF-8 | 10.0±0.0 | 15.7±0.9 | BF-9 | 13.7±0.5 | NA | 10.3±0.5 |
| LF-11 | NA | 11.7±0.5 | BF-10 | 12.0±0.0 | NA | 9.0±0.0 |
| LF-12 | 11.3±0.5 | 17.0±1.6 | Chloramphenicol | 29.0±0.8 | 10.0±0.0 | 33.3±1.2 |
| LF-13 | NA | 11.0±0.0 |  | | | |
| LF-16 | 14.3±0.5 | - |  |  |  |  |
| LF-17 | 11.7±0.5 | - |  |  |  |  |
| Chloramphenicol | 29.0±0.8 | 33.3±1.2 |  |  |  |  |

Here, values are mean ± SD, n= 3, NA = No activity.

**LC-MS based chemical characterization of the extracts**

| (a) L-ETA   | (b) L-MET   |
| --- | --- |
| (c) L-AQU   | (d) L-WAI   |

**Supplementary Fig. 7** NPC super classes, classes, and subclasses for the identified features in *A. dealbata* leaf extracts. Here, NPC (natural product classification) superclass, class, and sub-class for the identified features were calculated by taxonomically informed metabolite annotation (TIMA) of the LC-MS/MS data. Sunburst plots present the number of features identified by TIMA without any quantitative information. Different levels of the graph present superclass (inner circle), class (middle circle), and sub-class (outer circle) of the same feature.

| (a) B-ETA   | (b) B-MET   |
| --- | --- |
| (c) B-AQU   | (d) B-WAI   |

**Supplementary Fig. 8** NPC super class, class, and subclasses for the identified features in *A. dealbata* bark extracts. Here, NPC (natural product classification) superclass, class, and sub-class for the identified compounds were calculated by taxonomically informed metabolite annotation (TIMA) of the LC-MS/MS data. Sunburst plots present the number of features identified by TIMA without any quantitative information. Different levels of the graph present superclass, class, and sub-class of the same feature.

(a)

(b)

**Supplementary Fig. 9:** Feature-based molecular networking (FBMN) of the leaf (a) and bark (b) extracts generated from positive LC–MS/MS data. Here clusters were numbered for further visualisation and discussion in the manuscript.

**Supplementary Table 5**: Some tentatively identified compounds from *A. dealbata* leaf and bark extracts with GNPS-based library annotation using positive and negative ionization in LC-MS/MS.

| **Precursor m/z** | **Adduct** | **Name** | **Structure** | **Cosine score** | **Tissue type** |
| --- | --- | --- | --- | --- | --- |
| 163.039 | [M+H-H2O]^+^ | Caffeic acid |  | 0.80 | Bark |
| 257.081 | [M+H]^+^ | 1-(2,4-dihydroxyphenyl)-3-(4-hydroxyphenyl)prop-2-en-1-one |  | 0.78 | Bark |
| 271.06 | [M+H]^+^ | Apigenin |  | 0.99 | Leaf, Bark |
| 273.076 | [M+H]^+^ | Naringenin |  | 0.98 | Bark |
| 287.055 | [M+H]^+^ | Kaempferol |  | 0.92 | Leaf, Bark |
| 287.055 | [M+H]^+^ | Luteolin |  | 0.97 | Leaf, Bark |
| 289.072 | [M-H]^-^ | Catechin |  | 0.98 | Leaf, Bark |
| 294.179 | [M-H]^-^ | 6-Gingerol |  | 0.82 | Leaf |
| 297.119 | [M-H]^-^ | Rhodioloside |  | 0.98 | Leaf, Bark |
| 303.050 | [M+H]^+^ | Quercetin |  | 0.92 | Leaf, Bark |
| 307.081 | [M+H]^+^ | Epigallocatechin |  | 0.91 | Bark |
| 315.051 | [M-H]^-^ | Platyphyllonol |  | 0.75 | Bark |
| 317.065 | [M+H]^+^ | 3-O-Methylquercetin |  | 0.87 | Leaf, Bark |
| 318.155 | [M+NH4]^+^ | Salidroside |  | 0.93 | Leaf |
| 319.045 | [M+H]^+^ | Myricetin |  | 0.94 | Leaf, Bark |
| 325.049 | [M+Cl]^+^ | Cianidanol |  | 0.83 | Bark |
| 344.228 | [M+NH4]^+^ | 3-[4-[3,4,5-trihydroxy-6-(hydroxymethyl)oxan-2-yl]oxyphenyl]prop-2-enoic acid |  | 0.85 | Leaf |
| 355.102 | [M+H]^+^ | Cryptochlorogenic acid |  | 0.95 | Leaf |
| 383.061 | [M+H]^+^ | 4-hydroxy-3-(4-methoxy -6-methyl-5,6,7,8-tetrahydro-[1,3]dioxolo[4,5-g]isoquinolin-5-yl)-chromen-2-one |  | 0.93 | Leaf |
| 417.119 | [M-H]^-^ | ST077156 |  | 0.92 | Bark |
| 430.378 | [M+H]^+^ | α-Tocopherol |  | 0.80 | Leaf |
| 433.078 | [M+H]^+^ | Quercetin-3-arabinoside |  | 0.87 | Bark |
| 433.114 | [M-H]^-^ | Naringenin-7-O-glucoside |  | 0.91 | Leaf |
| 435.13 | [M-H]^-^ | [3-hydroxy-4-[3,4,5-trihydroxy-6-(hydroxymethyl)oxan-2-yl]oxyphenyl]methyl 3,4-dihydroxybenzoate |  | 0.83 | Leaf, Bark |
| 448.218 | [M+NH4]^+^ | 2-Phenylethyl rutinoside |  | 0.88 | Leaf |
| 449.108 | [M+H]^+^ | Kaempferol-3-O -glucoside |  | 0.94 | Leaf, Bark |
| 453.139 | [M+H]^+^ | NCGC00385785-01 |  | 0.85 | Bark |
| 461.109 | [M-H]^-^ | 5-hydroxy-2-(4-hydroxy-3-methoxyphenyl) -7-[3,4,5-trihydroxy-6-(hydroxymethyl)( 2H-3,4,5,6-tetrahydropyran-2-yloxy)]chromen-4-one |  | 0.80 | Leaf |
| 463.088 | [[M+H]^+^ | Hyperoside |  | 0.77 | Leaf |
| 463.088 | [M-H]^-^ | Isoetin O-glucopyranoside |  | 0.84 | Bark |
| 465.104 | [M-H]^-^ | Taxifolin-3-glucoside |  | 0.90 | Leaf |
| 465.103 | [M+H]^+^ | Quercetin-3-O-galactoside |  | 0.90 | Leaf |
| 481.097 | [M+H]^+^ | Myricetin 3-O -galactoside |  | 0.89 | Leaf, Bark |
| 521.203 | [M-H]^-^ | Isolariciresinol 4-O-glucopyranoside |  | 0.76 | Leaf |
| 536.165 | [M+K]^+^ | 3,4-di-O-galloylquinic acid |  | 0.87 | Leaf, Bark |
| 540.244 | [M+NH4]^+^ | Lariciresinol 4-O-glucoside |  | 0.87 | Leaf |
| 579.137 | [M-H]^-^ | 2-(3,4-dihydroxyphenyl)-5-hydroxy-7-[3,4,5-trihydroxy-6-[(3,4,5-trihydroxyoxan-2-yl)oxymethyl]oxan-2-yloxy]chromen-4-one |  | 0.82 | Leaf |
| 579.149 | [M+H]^+^ | Procyanidin B2 |  | 0.90 | Bark |
| 595.165 | [M+H]^+^ | Kaempferol-7- neohesperidoside |  | 0.93 | Leaf |
| 611.16 | [M+H]^+^ | Rutin |  | 0.94 | Leaf, Bark |
| 625.141 | [M-H]^-^ | Myricetin 3-O-rutinoside |  | 0.79 | Leaf |
| 661.118 | [M+Cl]^-^ | Narcissoside |  | 0.79 | Leaf |
| 684.203 | [M+K]^+^ | 6-Methoxycoumarin 7-O-dirhamnopyranoside |  | 0.84 | Bark |
| 741.223 | [M+H]^+^ | Mauritianin |  | 0.88 | Leaf |
| 757.218 | [M+H]^+^ | Manghaslin |  | 0.88 | Leaf, Bark |
| 895.194 | [2M-H]^-^ | Luteolin-7-O-glucoside |  | 0.95 | Leaf |
| 901.263 | [M-H]^-^ | NCGC00380496-01 |  | 0.81 | Leaf |

In the table, the compounds were tentatively identified by GNPS-based library annotations in either positive or negative mode LC-MS/MS, the respective adduct represents their ionization mode. The cosine score represents the degree of similarity between the experimental MS/MS spectrum and the corresponding reference library spectrum, based on the alignment of fragment ions and their relative intensities. The score ranges from 0 to 1, with higher values indicating greater spectral similarity (Wang et al., 2016). For example, a cosine score of 0.85 indicates a strong spectral match with the library spectrum. Only annotations with a minimum cosine score of 0.7 were taken into consideration. All structures were taken from the respective GNPS databases.

**Supplementary Table 6**: Identification and quantification of polyphenols using standards in targeted LC-MS.

| **Samples** | **Epigallocatechin** | **Catechin** | **Procyanidin B2** | **Rutin** | **Naringenin** | **Myricetin** | **Luteolin** | **Quercetin** | **Apigenin** |
| --- | --- | --- | --- | --- | --- | --- | --- | --- | --- |
| µg/mg of the samples | | | | | | | | | |
| B-ETA | 1.03 | 24.6 | 69.1 | 1.91 | - | 0.86 | - | 0.04 | TA |
| BF-1 | - | - | - | 0.04 | - | - | - | - | TA |
| BF-2 | - | - | - | - | - | - | - | - | - |
| BF-3 | 1.33 | 144 | 22.4 | 0.05 | - | - | - | - | TA |
| BF-4 | 0.12 | 14.9 | 252 | 0.06 | - | 0.86 | - | 0.06 | TA |
| BF-5 | 0.47 | 0.08 | 17.9 | 3.08 | - | - | - | 0.04 | TA |
| BF-6 | 0.48 | 0.06 | 0.26 | 0.07 | - | 0.01 | - | 0.04 | TA |
| BF-7 | - | 0.01 | 0.17 | 0.04 | - |  | - | 0.04 | - |
| BF-8 | - | - | 0.17 | 0.04 | - | - | - | - | TA |
| BF-9 | - | - | - | - | - | - | - | - | - |
| BF-10 | - | - | - | - | - | - | - | - | - |
| L-MET | 0.47 | 0.31 | 5.12 | 33.2 | 0.08 | 0.73 | 0.25 | 0.06 | TA |
| LF-1 | - | - | 0.17 | 19.73 | - | 0.86 | 0.09 | 0.07 | - |
| LF-2 | - | - | 0.17 | - | - | - | - | 0.04 | - |
| LF-3 | - | - | - | 0.08 | 0.07 | - | - | - | - |
| LF-4 | 0.48 | 0.82 | 0.77 | 0.13 | - | - | - | 0.04 | - |
| LF-5 | - | - | - | 0.04 | - | 0.01 | - | - | - |
| LF-6 | - | - | - | - | - | - | - | - | - |
| LF-7 | - | - | - | - | - | - | - | - | - |
| LF-8 | - | - | - | 0.04 | 0.08 | - | - | - | - |
| LF-9 | - | - | 0.19 | 57.9 | - | 0.87 | - | 0.06 | - |
| LF-10 | - | - | - | 0.04 | - | - | - | - | - |
| LF-11 | - | - | - | - | - | - | - | - | - |
| LF-12 | - | - | - | - | 0.08 | - | - | - | - |
| LF-13 | - | 0.10 | 0.18 | 0.20 | 0.07 | 0.87 | - | 0.05 | - |
| LF-14 | - | - | 0.18 | 0.10 | - | 0.87 | 0.06 | 0.06 | - |
| LF-15 | - | - | - | - | - | 0.85 | - | - | - |
| LF-16 | - | - | - | - | - | - | - | - | - |
| LF-17 | - | - | - | - | - | - | - | - | - |

Data represents targeted identification and quantification of 11 polyphenolic compounds in the extracts and fractions, where genistein and isorhamnetin were not detected in any of the samples and eliminated from the table. LF represents leaf fraction, while BF represents bark fractions with the respective fraction numbers, TA = trace amount (< 0.05 µM). Empty cells in the table represent absence of compounds in the respective samples.

**NMR-based characterization of the bioactive fractions**

**Supplementary Table 7:** Summary of the acquisition parameters used for the ^1^H, ^13^C, COSY, HSQC and HMBC NMR experiments, including pulse sequences, spectral widths, number of scans (NS), and optimised heteronuclear coupling constants where applicable.

| **Experiment** | **Pulse Sequence** | **Spectral Width (ppm)** | **No. of Scans (NS)** | **Coupling constant** |
| --- | --- | --- | --- | --- |
| ^1^H | zg | 15 | 8 | - |
| ^13^C | zgpg30 | 200 | 11264 | - |
| COSY | cosygpmfphpp | 13 | 32 | - |
| HSQC | hsqcedetgpsisp2.3 | 12/180 | 32 | *^1^J*_CH_ = 145 Hz |
| HMBC | hmbcgplpndqf | 13/220 | 32 | ^n^*J*_CH_ = 10 Hz |

**NMRMine-Assisted Spin System Extraction**

To facilitate interpretation of complex NMR datasets, manually curated COSY peak lists and HSQC peak lists were exported from TopSpin as CSV files and processed using the NMRMine workflow. COSY peak lists were used to construct proton–proton connectivity networks, in which cross peaks were treated as edges linking correlated proton resonances. Connected components within the resulting network were extracted as tentative spin systems. HSQC peak lists were subsequently integrated to associate proton resonances within each spin system with their corresponding carbon chemical shifts.

The resulting spin systems were used as an organisational tool to highlight recurring correlation patterns and assist class-level structural annotation. In particular, the workflow aided recognition of aromatic polyphenol-rich networks and oxygenated aliphatic/glycosidic networks within the bioactive fractions. Because signal overlap and peak-picking artefacts can generate implausible connections in complex mixtures, all NMRMine-derived spin systems were manually inspected against the original COSY and HSQC spectra. Final structural interpretation and compound-class assignments were therefore based on reproducible NMR correlations rather than automated outputs alone.

The NMRMine workflow was used solely to organise COSY and HSQC correlations into tentative spin systems and does not provide automated compound identification or definitive structural assignments.

**Supplementary Table 8**: NMRMine-derived spin systems extracted from the combined COSY and HSQC datasets for (a) BF-4, (b) BF-5, (c) BF-6, and (d) LF-13. Each spin system (SS) comprises correlated ^1^H resonances identified from COSY connectivity and their associated ^13^C chemical shifts assigned from HSQC spectra. Spin system numbering (SS1, SS2, etc.) is consistent with that used in the main text to facilitate structural interpretation and class-level metabolite annotation. The workflow provides tentative spin-system organisation and does not constitute automated compound identification.

| **Spin System** | **^1^H ppm** | **^13^C ppm** |
| --- | --- | --- |
| a. **Spin System (SS) for BF-4** | | |
| **SS1** | 1.13, 1.15, 1.16, 1.19, 1.81, 1.84, 1.86, 1.87, 1.9, 1.91, 1.93, 1.98, 2.0, 2.02, 2.09, 2.11, 2.12, 2.13, 2.16, 2.17, 2.18, 2.19, 2.41, 2.46, 2.49, 2.5, 2.51, 2.52, 2.54, 2.55, 2.56, 2.58, 2.59, 2.61, 2.63, 2.64, 2.65, 2.66, 2.68, 2.69, 2.73, 2.75, 2.76, 2.77, 2.78, 2.79, 2.8, 2.81, 2.83, 2.85, 2.87, 2.88, 2.9, 3.09, 3.33, 3.41, 3.43, 3.59, 3.62, 3.63, 3.64, 3.68, 3.71, 3.72, 3.73, 3.75, 3.76, 3.78, 3.81, 3.82, 3.92, 3.94, 3.95, 3.96, 3.97, 3.99, 4.0, 4.01, 4.03, 4.04, 4.05, 4.06, 4.07, 4.08, 4.1, 4.13, 4.14, 4.36, 4.37, 4.43, 4.44, 4.49, 4.51, 4.55, 4.57, 4.59, 4.62, 4.65, 4.67, 4.71, 4.72, 4.74, 4.75, 4.76, 4.78, 4.79, 4.8, 4.81, 4.83, 4.85, 4.86, 5.17, 5.19, 5.22, 5.23, 5.49, 7.6, 7.69, 7.73, 10.74 | 17.9, 26.5, 26.7, 27.0, 31.2, 40.4, 40.7, 46.0, 47.9, 48.8, 49.3, 50.8, 67.1, 67.3, 67.6, 67.8, 69.6, 69.7, 75.4, 79.2, 79.4, 81.0, 81.2, 81.3, 81.7, 83.3 |
| **SS2** | 1.37, 1.61, 1.7, 3.01 |  |
| **SS3** | 3.69, 4.52, 4.61 | 40.4, 67.6, 83.3 |
| **SS4** | 1.2, 3.67 | 67.6 |
| **SS5** | 5.94, 5.96, 5.98, 6.0, 6.02, 6.03, 6.19, 6.2, 6.22, 6.24, 6.25, 6.26, 6.28, 6.3, 6.32, 6.34, 6.36, 6.38, 6.4, 6.47, 6.5, 6.52, 6.53, 6.56, 6.57, 6.59, 6.61, 6.63, 6.64, 6.66, 6.68 | 95.0, 95.9, 96.1, 102.1, 102.2, 103.6, 103.8, 104.9, 105.0, 105.2, 105.4, 105.6, 105.8, 106.1, 106.3, 106.8, 107.0, 107.7, 107.9, 113.3, 114.2, 114.5, 114.9, 118.1, 119.3, 128.6, 128.8, 129.8 |
| **SS6** | 0.92, 1.3, 1.38 | 29.1 |
| **SS7** | 2.6, 4.09 | 67.3 |
| **SS8** | 6.67, 6.75 | 114.9, 118.6 |
| **SS9** | 5.13, 8.55, 8.57 |  |
| **SS10** | 6.08, 6.12 | 94.5, 105.8 |
| **SS11** | 5.91, 8.56 |  |
| **SS12** | 6.78, 6.85 | 114.0, 114.7, 119.8 |
| b. **Spin System (SS) for BF-5** | | |
| **SS1** | 1.15, 1.16, 1.18, 1.2, 1.22, 1.23, 1.36, 1.61, 1.62, 1.66, 1.67, 1.69, 1.79, 1.81, 1.82, 1.83, 1.84, 1.85, 1.86, 1.87, 1.88, 1.89, 1.9, 1.91, 1.93, 1.95, 1.96, 1.97, 1.98, 1.99, 2.0, 2.01, 2.02, 2.03, 2.04, 2.05, 2.06, 2.07, 2.08, 2.09, 2.11, 2.12, 2.13, 2.14, 2.15, 2.16, 2.17, 2.18, 2.19, 2.2, 2.21, 2.22, 2.23, 2.24, 2.25, 2.26, 2.27, 2.28, 2.3, 2.35, 2.36, 2.37, 2.4, 2.41, 2.42, 2.43, 2.44, 2.45, 2.46, 2.47, 2.48, 2.49, 2.5, 2.51, 2.52, 2.53, 2.54, 2.55, 2.56, 2.57, 2.58, 2.59, 2.6, 2.61, 2.62, 2.63, 2.64, 2.65, 2.66, 2.67, 2.68, 2.69, 2.7, 2.71, 2.72, 2.73, 2.74, 2.75, 2.76, 2.77, 2.78, 2.79, 2.8, 2.81, 2.82, 2.83, 2.84, 2.85, 2.86, 2.87, 2.88, 2.89, 2.9, 2.91, 2.92, 2.93, 2.94, 2.96, 2.97, 2.99, 3.0, 3.01, 3.02, 3.04, 3.05, 3.06, 3.08, 3.11, 3.35, 3.38, 3.39, 3.4, 3.41, 3.44, 3.45, 3.49, 3.5, 3.51, 3.52, 3.54, 3.55, 3.57, 3.58, 3.59, 3.6, 3.61, 3.62, 3.63, 3.64, 3.66, 3.68, 3.69, 3.71, 3.72, 3.73, 3.75, 3.76, 3.78, 3.8, 3.81, 3.83, 3.9, 3.94, 3.95, 3.96, 3.97, 3.99, 4.0, 4.01, 4.03, 4.04, 4.05, 4.06, 4.07, 4.08, 4.09, 4.1, 4.15, 4.37, 4.38, 4.39, 4.41, 4.42, 4.43, 4.44, 4.46, 4.48, 4.5, 4.51, 4.55, 4.56, 4.57, 4.58, 4.59, 4.6, 4.62, 4.63, 4.64, 4.65, 4.69, 4.7, 4.71, 4.72, 4.73, 4.74, 4.75, 4.76, 4.78, 4.79, 4.8, 4.81, 4.82, 4.83, 4.84, 4.85, 4.86, 4.87, 4.88, 4.89, 4.9, 4.92, 4.94, 4.95, 4.96, 4.97, 4.98, 4.99, 5.0, 5.14, 5.16, 5.17, 5.19, 5.21, 5.22, 5.25, 5.27, 5.28, 7.61, 10.78 | 27.0, 27.2, 27.4, 27.6, 27.9, 28.1, 31.4, 31.6, 34.9, 35.1, 35.6, 35.8, 36.0, 67.3, 68.1, 71.8, 75.4, 79.0, 79.2, 81.0, 81.2, 81.3, 81.5, 83.1, 83.3 |
| **SS2** | 5.13, 5.91, 8.56 |  |
| **SS3** | 0.94, 5.93, 5.94, 5.96, 5.98, 6.0, 6.17, 6.19, 6.2, 6.21, 6.22, 6.24, 6.25, 6.26, 6.28, 6.29, 6.3, 6.31, 6.32, 6.33, 6.34, 6.35, 6.36, 6.38, 6.43, 6.44, 6.47, 6.49, 6.52, 6.54, 6.56, 6.58, 6.59, 6.6, 6.61, 6.62, 6.63, 6.64, 6.65, 6.67, 6.7, 6.71, 6.73, 6.74, 6.75, 6.76, 6.81, 6.82, 6.84, 6.88, 6.91, 10.03, 10.14, 10.37, 11.37, 11.39, 11.6, 11.63, 11.69, 11.72 | 95.2, 96.1, 101.9, 102.1, 102.2, 102.4, 102.6, 103.5, 103.6, 103.8, 104.7, 105.0, 105.2, 105.4, 106.3, 106.5, 106.8, 107.0, 107.5, 107.9, 113.5, 113.8, 114.5, 114.9, 118.2, 118.6, 128.4, 128.6, 128.8, 128.9, 129.8 |
| **SS4** | 0.89, 0.92, 1.3, 1.35 | 29.1 |
| **SS5** | 1.52, 6.77, 9.45 | 113.5, 114.7, 118.6 |
| **SS6** | 1.51, 6.78, 9.47, 9.53 | 114.7, 118.6 |
| **SS7** | 0.87, 0.95, 1.32 | 29.1 |
| **SS8** | 1.31, 7.38 | 29.1 |
| **SS9** | 3.09, 3.42, 3.86 |  |
| **SS10** | 6.53, 6.72 | 105.4, 106.8, 114.5, 114.9, 128.6 |
| c. **Spin System (SS) for BF-6** | | |
| **SS1** | 0.93, 1.35 | 13.0 |
| **SS2** | 0.88, 0.9, 1.3 | 29.1 |
| **SS3** | 1.18, 3.61 | 68.0 |
| **SS4** | 1.22, 3.66 |  |
| **SS5** | 1.63, 1.64, 1.7, 1.81, 1.82, 1.83, 1.84, 1.86, 1.88, 1.89, 1.9, 1.93, 1.94, 1.96, 1.97, 1.98, 1.99, 2.0, 2.01, 2.02, 2.05, 2.12, 2.15, 2.17, 2.18, 2.19, 2.21, 2.22, 2.23, 2.24, 2.25, 2.28, 2.29, 2.3, 2.31, 2.36, 2.38, 2.41, 2.44, 2.45, 2.46, 2.47, 2.48, 2.49, 2.5, 2.51, 2.52, 2.53, 2.54, 2.55, 2.56, 2.58, 2.59, 2.6, 2.61, 2.62, 2.63, 2.64, 2.65, 2.66, 2.71, 2.73, 2.74, 2.75, 2.76, 2.77, 2.78, 2.79, 2.8, 2.81, 2.82, 2.84, 2.85, 2.86, 2.88, 2.89, 2.91, 2.92, 2.94, 2.96, 2.98, 2.99, 3.0, 3.03, 3.04, 3.05, 3.99, 4.05, 4.06, 4.08, 4.59, 4.62, 4.65, 4.74, 4.75, 4.76, 4.79, 4.8, 4.81, 4.82, 4.83, 4.85, 4.86, 4.87, 4.88, 4.89, 4.9, 4.91, 4.93, 4.95, 4.96, 4.98, 4.99 | 26.7, 26.9, 27.2, 27.4, 27.9, 31.4, 31.5, 32.5, 32.7, 34.9, 35.1, 35.3, 35.5, 35.6, 35.8, 36.2, 67.2, 73.3, 73.4, 79.0, 81.2, 81.4 |
| **SS6** | 3.59, 4.4, 4.44 | 72.1, 80.9, 83.2 |
| **SS7** | 3.67, 4.45 | 83.0 |
| **SS8** | 2.03, 4.7 |  |
| **SS9** | 3.5, 4.43 | 72.1, 80.9, 83.2 |
| **SS10** | 3.97, 4.58 | 73.4, 81.2 |
| **SS11** | 2.68, 2.95, 3.02, 4.09 | 27.9 |
| **SS12** | 3.27, 3.85 |  |
| **SS13** | 1.6, 1.66, 3.21, 3.83 | 55.3, 67.6 |
| **SS14** | 2.04, 2.26 |  |
| **SS15** | 5.94, 6.34, 6.58, 6.74 | 104.9, 118.3, 118.5, 128.5 |
| **SS16** | 6.47, 6.9 |  |
| **SS17** | 6.23, 6.35, 6.56, 6.89, 7.12 | 104.9, 105.3, 106.9, 113.7, 128.5 |
| **SS18** | 5.93, 5.99, 6.0, 6.18, 6.24, 6.25, 6.26, 6.3, 6.31, 6.33, 6.45, 6.61, 6.62, 6.63, 6.66, 6.67, 6.7, 6.71, 6.75, 6.77 | 102.6, 104.9, 106.4, 108.1, 114.9, 118.5, 128.4, 128.6 |
| **SS19** | 6.2, 6.41, 6.52, 6.82 | 114.6 |
| **SS20** | 6.21, 6.29, 6.49, 6.59, 7.11 | 102.6, 105.2, 108.1, 128.5 |
| **SS21** | 6.43, 6.92 |  |
| **SS22** | 6.38, 6.87 | 113.7 |
| **SS23** | 6.42, 6.91 |  |
| **SS24** | 5.95, 6.28, 6.64 | 108.1, 114.9, 118.3, 128.4 |
| **SS25** | 6.32, 6.53, 6.68 | 102.6, 105.3, 106.9, 114.6 |
| **SS26** | 5.07, 5.96, 8.5, 8.54 | 118.3 |
| **SS27** | 1.36, 1.67, 1.75, 3.15 |  |
| **SS28** | 1.73, 3.16 |  |
| **SS29** | 2.16, 2.42, 3.38 | 36.2 |
| **SS30** | 2.57, 4.0, 4.6, 4.66, 4.94 | 26.9, 31.5, 31.7, 32.5, 73.4, 81.2 |
| d. **Spin System (SS) for LF-13** | | |
| **SS1** | 3.2, 5.32 |  |
| **SS2** | 1.02, 3.42, 3.54, 3.63 | 69.8 |
| **SS3** | 0.77, 0.88, 1.01, 1.04, 1.09, 1.25, 1.27, 2.54, 2.92, 3.05, 3.15, 3.41, 3.45, 3.69, 7.51, 7.85 | 22.5, 25.3, 29.3, 31.8, 56.5, 70.6, 77.1, 77.4 |
| **SS4** | 6.86, 7.98 |  |
| **SS5** | 6.91, 8.0 |  |
| **SS6** | 6.87, 7.97 |  |
| **SS7** | 0.95, 2.16 |  |
| **SS8** | 1.18, 3.28, 3.52, 3.73, 3.82 |  |
| **SS9** | 1.29, 1.37, 1.49, 1.53, 2.31, 2.46 | 22.5, 24.7 |
| **SS10** | 1.2, 3.5, 3.62 | 69.8 |
| **SS11** | 1.07, 3.35, 3.36, 3.48, 3.67, 3.89, 4.03 | 61.3, 63.1, 65.9 |
| **SS12** | 0.98, 3.24 |  |
| **SS13** | 1.48, 1.56, 2.26, 2.27 | 33.9 |
| **SS14** | 0.99, 3.3, 3.33, 3.61 | 63.1 |
| **SS15** | 0.83, 1.3 |  |
| **SS16** | 0.82, 1.23 |  |
| **SS17** | 0.86, 8.3 |  |
| **SS18** | 3.06, 3.16 | 70.6, 77.1 |
| **SS19** | 2.82, 3.25 |  |
| **SS20** | 3.07, 3.26, 3.66, 3.92 | 61.3, 77.1 |
| **SS21** | 3.49, 3.76 | 56.1 |
| **SS22** | 3.59, 3.9 | 65.9 |
| **SS23** | 3.68, 4.05 | 61.3, 65.9 |
| **SS24** | 1.44, 2.28 | 33.9 |
| **SS25** | 1.55, 2.3 |  |
| **SS26** | 3.31, 3.43, 3.7 |  |
| **SS27** | 3.38, 3.64 | 69.8 |
| **SS28** | 1.22, 1.5 |  |

**Figure a:** ^1^H NMR spectrum of BF-4.

**Figure b:** ^13^C NMR spectrum of BF-4.

**Figure c:** COSY spectrum of BF-4.

**Figure d:** HSQC spectrum of BF-4.

**Figure e:** ^1^H NMR spectrum of BF-5.

**Figure f:** ^13^C NMR spectrum of BF-5.

**Figure g:** COSY spectrum of BF-5.

**Figure h:** HSQC spectrum of BF-5.

**Figure i:** ^1^H NMR spectrum of BF-6.

**Figure j:** ^13^C NMR spectrum of BF-6.

**Figure k:** COSY spectrum of BF-6.

**Figure l:** HSQC spectrum of BF-6.

**Figure m:** ^1^H NMR spectrum of LF-13.

**Figure n:** ^13^C NMR spectrum of LF-13.

**Figure o:** COSY spectrum of LF-13.

**Figure p:** HSQC spectrum of LF-13.

**Figure q:** HMBC spectrum of LF-13.

**Supplementary Fig. 10** NMR spectrum of BF-4 (a-d), BF-5 (e-h), BF-6 (i-l) and LF-13 (m-q).

**References**

Wang, M., Carver, J. J., Phelan, V. V., Sanchez, L. M., Garg, N., Peng, Y., Nguyen, D. D., Watrous, J., Kapono, C. A., Luzzatto-Knaan, T., Porto, C., Bouslimani, A., Melnik, A. V., Meehan, M. J., Liu, W.-T., Crüsemann, M., Boudreau, P. D., Esquenazi, E., Sandoval-Calderón, M., . . . Bandeira, N. (2016). Sharing and community curation of mass spectrometry data with Global Natural Products Social Molecular Networking. *Nature Biotechnology*, *34*(8), 828-837. <https://doi.org/10.1038/nbt.3597>
